# Morphological and dietary changes encoded in the genome of *Beroe ovata*, a ctenophore-eating ctenophore

**DOI:** 10.1093/nargab/lqae072

**Published:** 2024-06-18

**Authors:** Alexandra M Vargas, Melissa B DeBiasse, Lana L Dykes, Allison Edgar, T Danielle Hayes, Daniel J Groso, Leslie S Babonis, Mark Q Martindale, Joseph F Ryan

**Affiliations:** Whitney Laboratory for Marine Bioscience, University of Florida, St. Augustine, FL 32080, USA; Department of Biological Sciences, University of North Carolina at Charlotte, Charlotte, NC 28223, USA; Whitney Laboratory for Marine Bioscience, University of Florida, St. Augustine, FL 32080, USA; Department of Biology, Radford University, Radford, VA 24142, USA; Whitney Laboratory for Marine Bioscience, University of Florida, St. Augustine, FL 32080, USA; Whitney Laboratory for Marine Bioscience, University of Florida, St. Augustine, FL 32080, USA; Federated Department of Biological Sciences, New Jersey Institute of Technology, Newark, NJ 07102, USA; Whitney Laboratory for Marine Bioscience, University of Florida, St. Augustine, FL 32080, USA; Whitney Laboratory for Marine Bioscience, University of Florida, St. Augustine, FL 32080, USA; Computational Biology Program, Public Health Sciences Division, Fred Hutchinson Cancer Center, Seattle, WA 98109, USA; Human Biology Division, Fred Hutchinson Cancer Center, Seattle, WA 98109, USA; Department of Ecology and Evolutionary Biology, Cornell University, Ithaca, NY 14853, USA; Whitney Laboratory for Marine Bioscience, University of Florida, St. Augustine, FL 32080, USA; Department of Biology, University of Florida, Gainesville, FL 32611, USA; Whitney Laboratory for Marine Bioscience, University of Florida, St. Augustine, FL 32080, USA; Department of Biology, University of Florida, Gainesville, FL 32611, USA

## Abstract

As the sister group to all other animals, ctenophores (comb jellies) are important for understanding the emergence and diversification of numerous animal traits. Efforts to explore the evolutionary processes that promoted diversification within Ctenophora are hindered by undersampling genomic diversity within this clade. To address this gap, we present the sequence, assembly and initial annotation of the genome of *Beroe ovata*. *Beroe* possess unique morphology, behavior, ecology and development. Unlike their generalist carnivorous kin, beroid ctenophores feed exclusively on other ctenophores. Accordingly, our analyses revealed a loss of chitinase, an enzyme critical for the digestion of most non-ctenophore prey, but superfluous for ctenophorivores. Broadly, our genomic analysis revealed that extensive gene loss and changes in gene regulation have shaped the unique biology of *B. ovata*. Despite the gene losses in *B. ovata*, our phylogenetic analyses on photosensitive opsins and several early developmental regulatory genes show that these genes are conserved in *B. ovata*. This additional sampling contributes to a more complete reconstruction of the ctenophore ancestor and points to the need for extensive comparisons within this ancient and diverse clade of animals. To promote further exploration of these data, we present BovaDB (http://ryanlab.whitney.ufl.edu/bovadb/), a portal for the *B. ovata* genome.

## Introduction

**Figure 1.**
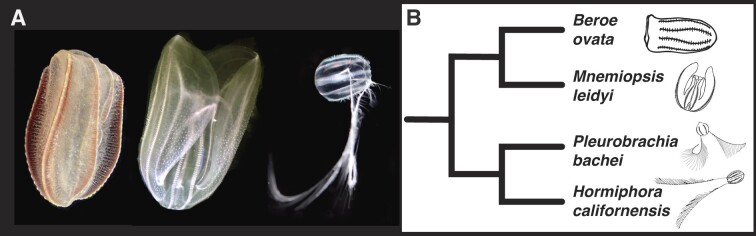
Ctenophore species and their relationships. (**A**) Three of the four ctenophore species included in this study. From left to right: *Beroe ovata*, *M. leidyi* and *P. bachei* (*H. californensis*, not pictured, is a tentaculate ctenophore with similar morphology to *P. bachei*). The photo of *B. ovata* was taken from an animal collected from St. Augustine, Florida (∼64 km north of the site where we collected animals for sequencing). (**B**) Cladogram showing the relationship between all four ctenophore species included in this study based on (31–33). Photo credits: *Beroe*: Joseph Ryan; *Mnemiopsis*: Arianna Rodriguez, used with permission; *Pleurobrachia*: Joseph Ryan.

Ctenophores are a powerful and emerging model system for studying development, regeneration, evolutionary biology and ecology (1). They have been attractive for studies in embryonic and adult tissue development since the 19th century (2–10) and possess several traits found in bilaterians, including a through-gut, nervous system and muscles. Ctenophores are characterized by eight longitudinal rows of motile cilia called ctenes that are arranged along the oral/aboral axis of their bodies that they use for swimming. Their ciliary beating and locomotion are controlled by their aboral organ, and most species use tentacles lined with adhesive cells called colloblasts to capture planktonic prey (primarily crustaceans). While ctenes, aboral organs and tentacles with colloblasts are ancestral traits in Ctenophora, the morphology and function of these features have diversified as ctenophores have invaded and adapted to novel niche spaces. Across Ctenophora, there is considerable variation in morphology, habitat use and diet, yet ctenophores continue to be largely neglected when it comes to intraphylum comparative genomic studies. Out of ∼200 described species (11), there are currently only four published ctenophore genomes, each from *Mnemiopsis leidyi*, *Pleurobrachia bachei*, *Hormiphora californensis* and *Bolinopsis microptera* (12–15) (Figure 1). The majority of subsequent genomic studies on ctenophores have focused on *M. leidyi* due to the availability of a well-maintained set of genomic resources (16–18), easy access to embryos, their high regenerative capacity (19–21) and their widespread occurrence along the Atlantic coast of the Americas and several Eurasian seas (22–26).

Among the diversity of described ctenophores, the genus *Beroe* is unique in its morphology, feeding behavior and diet. *Beroe* lack tentacles throughout their lives and feed raptorially on a diet that consists almost exclusively of other ctenophores, which they can detect via chemical stimulation (22,27). They swim mouth-forward to seek their prey, and possess adhesive cells that keep their mouths closed while swimming (28), a behavior that differs from all other ctenophores. While *Beroe* can regenerate their lips (29,30), they lack the whole-body regenerative abilities found in *Mnemiopsis*. Molecular-based ctenophore phylogenies place *Beroe* within the rest of ctenophores, as opposed to sister to the rest of ctenophores (31–34). The most parsimonious explanation for this phylogenetic position is that beroids lost tentacles and that their unusual feeding behavior is derived (35). *Beroe* has thus gained a new diet and behavioral regime, and at the same time lost complex organs and cell types (e.g. colloblasts). Therefore, it is instructive to determine whether these gains and losses are found in the genome.

Here, we have sequenced, assembled and annotated the genome of *B. ovata* Bruguière, 1789. To investigate the evolutionary genomic changes that led to the unique morphology, feeding behavior and diet of *B. ovata*, we conducted phylogenetic analyses and searched for genes associated with diet. Our phylogenetic analyses comprised developmental and light-sensing genes from *B. ovata* and other ctenophores. Based on findings that the lineage leading to *Beroe* had lost >200 genes associated with tentacles (35), we anticipated identifying additional gene losses in the *B. ovata* genome resulting from the evolutionary loss of tentacles and colloblasts, as well as its shift to a distinct ctenophorivorous diet. We also present transcriptomic data sequenced from the comb rows and aboral organs from *B. ovata*, where we performed comparative analyses to *M. leidyi*. We identify highly expressed tissue-specific genes shared by the last common ancestor of *B. ovata* and *M. leidyi*. Taken together, our study provides insights into the genomic and transcriptomic dynamics within ctenophores and provides a resource for future ctenophore genomic analyses.

## Materials and methods

### Transparency and reproducibility

Prior to conducting all phylogenetic analyses, we wrote and published a phylotocol (36) on GitHub that outlined our planned analyses. Any methodological deviations or changes during the course of the study have been outlined and justified in the current version of the phylotocol. These adjustments are available along with alignments, trees, commands and custom scripts used in this study at https://github.com/josephryan/Beroe_genome (referred to as the project GitHub repo throughout). Custom scripts for genome assembly, annotation and analysis of tissue-specific transcriptomic data are also available in the project GitHub repo. An archived version of the project GitHub repo is available at doi:10.5281/zenodo.10794950.

### Animal collection and genome sequencing

We carefully collected *B. ovata* adults from Port Orange, Florida (29°08′50.6″N, 80°58′35.7″W) during March 2015 using a cteno-dipper (i.e. a plastic beaker attached to a broom handle) to prevent injuring the animals. We extracted genomic DNA from a single adult individual using DNAzol and sent it to the University of Washington Genome Sciences Pacific Biosciences (PacBio) Facility for library preparation and continuous long-read (CLR) sequencing. Library preparation was carried out using the Template Prep Kit v1, and sequencing was performed on the PacBio RSII with eight SMRT cells. We also sequenced genomic DNA from one adult using 150-bp paired-end sequencing on the Illumina HiSeq 2500 platform at the University of Texas, Austin Genomic Sequencing and Analysis Facility. Details on sequencing are available in Table 1.

**Table 1:** . Summary of genomic and transcriptomic data sequenced for this study

Data type	Accession numbers	Reads	Total Gb	Sequencing machine
Genomic DNA	ERR2206326, ERR2206328	27 218 713 (paired-end)	8.2	Illumina HiSeq 2500
Genomic DNA	ERR9829220	156 005	1.3	PacBio RSII
Genomic DNA	ERR9829408	143 820	1.1	PacBio RSII
Genomic DNA	ERR9829405	165 986	1.4	PacBio RSII
Genomic DNA	ERR9829409	144 846	1.3	PacBio RSII
Genomic DNA	ERR9814540	148 555	1.2	PacBio RSII
Genomic DNA	ERR9829410	163 381	1.3	PacBio RSII
Genomic DNA	ERR9829411	183 123	1.4	PacBio RSII
Genomic DNA	ERR12051222	163 887	1.3	PacBio RSII
RNA	ERR10009850	28 434 415 (paired-end)	8.5	Illumina NovaSeq 6000
RNA	ERR10006118	31 889 177 (paired-end)	9.6	Illumina NovaSeq 6000
RNA	ERR10009774	29 538 666 (paired-end)	8.8	Illumina NovaSeq 6000
RNA	ERR10016030	26 254 928 (paired-end)	7.9	Illumina NovaSeq 6000
RNA	ERR9993350	30 932 045 (paired-end)	9.3	Illumina NovaSeq 6000
RNA	ERR9993422	32 038 774 (paired-end)	9.6	Illumina NovaSeq 6000

### Genome assembly

To leverage the contiguity of long-read assemblies, while avoiding artifactual regional duplications (haplotigs) often associated with assemblies of older PacBio CLR sequencing (37), we implemented a strategy to scaffold an Illumina short-read assembly with artificial mate pairs generated from a PacBio long-read assembly. Below, we describe in further detail our assembly process for both the Illumina and PacBio sequence data, as well as our approach for scaffolding our Illumina short-read assembly.

To estimate the genome size for the adult we sequenced with Illumina, we counted canonical *k*-mers for *k* = 21 using Jellyfish (38) and estimated the genome size from the *k*-mer histogram using GenomeScope (39). We then removed adapters from the Illumina short reads using Trimmomatic v0.36 (40) and performed error correction with ErrorCorrectReads in AllPaths-LG v44837 (41). We assembled the Illumina short reads using Platanus v1.2.4 (42) with *k*-mer lengths of 31, 45 and 63. To identify the optimum assembly, we evaluated each using N50 and BUSCO metrics. BUSCO scores were generated based on a search against the eukaryotic database in BUSCO v5.1.2 (43) implemented in gVolante v2.0.0 (44). Based on these assessment statistics, we determined the *k* = 31 assembly to be superior to the *k* = 45 and *k* = 63 assemblies (Supplementary Table S1). To capture assembly information present in our suboptimal short-read assemblies (*k* = 45 and *k* = 63) that were not captured in our *k* = 31 assembly, we scaffolded the *k* = 31 assembly with *in silico* mate-pair libraries produced from the suboptimal assemblies. We used Matemaker v1.0.0 (https://github.com/josephryan/matemaker) to produce *in silico* mate-pair libraries of 2, 5, 10 and 15 kb insert sizes from the suboptimal assemblies. Using these mate-pair libraries, we scaffolded the optimal assembly (*k* = 31) using SSPACE v3.0 (45) and removed any reads <200 bp.

For PacBio long reads, we performed five assemblies in Canu v1.3 (46) using an estimated genome size of 89 Mb with a difference in overlap between corrected reads set to 0.075, 0.15 and 0.40, and an estimated genome size of 120 Mb with a difference in overlap between corrected reads set to 0.25 and 0.30, and corOutCoverage of 200. We adjusted these assembly parameters to correct for multiple haplotypes represented in the assembly. However, the resulting assemblies were nearly double the size of the *k* = 31 short-read assembly (Supplementary Table S1), and our BUSCO runs of these assemblies included many copies of BUSCO genes that were expected to be conserved single-copy genes. Together, these results suggested that we were capturing multiple independently assembled allelic contigs (haplotigs) in our Canu assemblies.

To leverage the lack of haplotigs in our short-read assembly and the superior contiguity of our long-read Canu assemblies, we used Matemaker to produce *in silico* mate-pair libraries of 2, 5, 10, 20, 50, 75 and 100 kb insert sizes from the Canu assemblies and used SSPACE to scaffold our Illumina short-read assembly described above with the *in silico* mate-pair libraries. We removed gaps from the scaffold that were >5 kb using the custom script break_big_gaps.pl (available in our project GitHub repo) and used Redundans v0.11c (47) to remove redundancy from the final assembly. We isolated and assembled scaffolds from the assembly that contained the mitochondrial genome. We also generated a preliminary annotation of this mitochondrial genome (see Supplementary Data). We then assessed our final assembly for repetitive content using RepeatModeler v2.0.4 (48) and RepeatMasker v.4.1.4 (www.repeatmasker.org/) through Dfam-TETools v3.7 (https://github.com/Dfam-consortium/TETools).

### Macrosynteny analysis

We analyzed conserved chromosome-scale synteny (macrosynteny) between *B. ovata* and *H. californensis*. To identify conserved genes, we performed diamond BLAST (49) to compute the reciprocal best BLAST hits between the *B. ovata* and *H. californensis* protein models using rbhXpress (https://github.com/SamiLhll/rbhXpress/tree/v1.2.3). Reciprocal best BLAST hits and genome annotations were used to identify significantly conserved macrosyntenic blocks in macrosyntR (50).

### Gene prediction

We predicted gene models with BRAKER v2.1.6 (51–53), which uses GeneMark-EX v4.68 (54–56) and AUGUSTUS v3.4.0 (57,58), by providing the genomic assembly and unassembled transcriptomic data aligned to the genomic assembly for training and prediction. We used STAR v.2.7.7a (59) to align unassembled transcriptomic reads to the genome assembly. We then sorted and converted the alignments to BAM format (60) using SAMtools v1.11 (61,62). These were used as input for gene predictions in BRAKER. Protein models were extracted using the getAnnoFasta.pl utility script in AUGUSTUS.

To check for potential missed gene predictions, we used OrthoFinder v2.23 (63) to build orthogroups from the following datasets: *B. ovata* protein models, *B. ovata* transcripts collected at 20 h post-fertilization (see Supplementary Data), *M. leidyi* ML2.2 protein models (12) and *H. californensis* Hcv1av93 protein models (14). We applied the custom script count_missed_genes.pl to identify candidate missed gene predictions. Orthogroups lacking *B. ovata* protein models but containing *B. ovata* transcripts and *H. californensis* and/or *M. leidyi* protein models were considered candidate missed gene predictions. We then ran the custom script confirm_missed_predictions.pl to confirm that *B. ovata* transcripts that were candidate missed gene predictions did not have significant BLASTP (64) hits (*E*-value <1e−6) to *B. ovata* gene models. Transcripts that did not have significant hits were translated into amino acid sequences using TransDecoder v.3.0.1 (github.com/TransDecoder) to identify coding regions with the single longest open reading frame. We aligned the coding regions to the *B. ovata* genomic scaffolds using BLAT (65). For each orthogroup, if there were one or more coding regions that aligned to more than one genomic scaffold, we ignored these due to the complexity of determining from which genomic region they originated. We combined the gene predictions from BRAKER and the missed gene predictions from AUGUSTUS, and determined the correct open reading frame for coding regions that did not have start codons using GffRead v.0.12.7 (66). Using this pipeline, we added 112 gene models that were not identified by BRAKER. Finally, we assessed the completeness of the genome, and the gene models using a search against the eukaryote database in BUSCO v5 and CEGMA v2.5 (67), implemented in gVolante (44).

### Gene family analysis

To assess the diversification of highly conserved developmental genes in three lineages of ctenophores with distinct morphologies and capacities for regeneration, we performed a phylogeny-based gene family analysis for transforming growth factor β (TGF-β) ligands, TGF-β receptors and Wnt ligands. We further explored the evolution of two gene families: opsins, which regulate light sensitivity, and chitinases, which are used to break down the chitinous exoskeletons of crustacean (and other non-ctenophore) prey. These genes were chosen because they are associated with the derived behavior and habitat use in *B. ovata*.

For phylogenetic analyses, we began by using hmmbuild from HMMR v3.3 (68) to construct hidden Markov models (HMMs) from published alignments that include sequences from *M. leidyi* of TGF-β ligands and TGF-β receptors [alignments from (69)], Wnt ligands (70) and opsins (71). We identified and aligned the *B. ovata* and *H. californensis* protein models to the TGF-β ligand, TGF-β receptor, Wnt ligand and opsin HMMs using hmm2aln.pl (https://github.com/josephryan/hmm2aln.pl). From our resulting alignment of Wnt ligands, we pruned the *Platynereis dumerilii* annelid Wnt9 sequence due to 91% gaps. We then generated a preliminary maximum-likelihood tree for each of the alignments using the multicore version of IQ-TREE v1.6.12 (72).

After conducting preliminary maximum-likelihood analyses on the TGF-β receptor and opsin alignments, we pruned sequences that were not members of focal clades (i.e. TGF-β receptors and opsins) since these families are part of larger gene superfamilies (i.e. TGF-β and GPCR superfamilies). We used the script make_subalignment (https://github.com/josephryan/make_subalignment) to exclude clades that did not include sequences from the starting published alignments (i.e. non-opsin G-protein-coupled receptors). We also removed duplicate isoforms and sequences with 281 or more gaps in the TGF-β receptor alignments and 184 or more gaps in opsin alignments. For the opsin sequence alignment, we removed *Mnemiopsis* opsin3 during the make_subalignment step as its inclusion led to the incorporation of a large clade of non-opsins, the vast majority of which lacked the critical lysine residue from helix VII, which is a defining feature of functional opsins (Supplementary Figures S1 and S2; see Supplementary Data for expanded analysis).

With all of these alignments, we performed a range of maximum-likelihood analyses using IQ-TREE and RAxML v8.2.12 (73) and applied the best-fit model identified by each program. For IQ-TREE analyses, we used default parameters. For analyses performed in RAxML, we used 25 random starting trees and 25 maximum parsimony starting trees. We compared the likelihood scores of the IQ-TREE analysis and the two RAxML analyses to select the best tree. For the best tree identified, we performed a bootstrap analysis with default settings in the program in which the tree was reconstructed to gain support values for relationships among sequences (in IQ-TREE the default was 1000 replicates and in RAxML the default was 100 replicates).

For our investigation of chitinase, we searched for evidence of chitinase genes in the *B. ovata* genome using a reciprocal best BLAST approach. The human genome encodes two true chitinases (chitotriosidase and acidic mammalian chitinase) and several chitinase-like proteins. Both classes of proteins have a conserved Glyco_hydro_18 domain (Pfam PF00704) and can bind chitin but only the true chitinases include a chitin-degrading catalytic site (74). We used BLASTP to search the two true human chitinase genes (NCBI accession numbers AAI03696.1 and NP_001244930.1) against the set of proteins predicted from the genomes of *B. ovata*, *M. leidyi* and *H. californensis*, extracted the top hits and searched the ctenophore proteins against the RefSeq database at NCBI restricting the organism query to *Homo sapiens* (taxID 9606). Ctenophore genes were determined to be proper chitinases only if they were the top hit for one of the human chitinase genes in both reciprocal BLAST searches. The same strategy was used to identify chitinase-like proteins from the three species of ctenophore using the human chitinase-like gene as a query (NP_001136146.1). In addition, we performed hmmscan using the default settings on the predicted proteomes of all three ctenophores and examined catalytic sites of any proteins with Glyco_hydro_18 domains that had an independent (domain-specific) *E*-value <0.05. Since these analyses were based on the quality of our gene annotation, we performed additional analyses using miniprot (75) to align identified ctenophore chitinase proteins to the *B. ovata* genome (see Supplementary Data). Finally, we also translated the *B. ovata* genome and used a Perl regular expression (m/FDG[^\*]?D[^\*]D[^\*]E/) to scan the translated genome for chitin-degrading catalytic sites [FDG(X)DXDXE; see fdgxdxdxe_in_genome.pl in the project GitHub repo].

### Comb row and aboral organ transcriptome sequencing

We sequenced tissue-specific transcriptomic data from *B. ovata* adults collected from the St. Augustine Inlet in St. Augustine, Florida. We isolated comb row and aboral organ tissues from three different individual adults (three biological replicates) and flash froze them on dry ice. RNA extraction, library preparation and sequencing were performed at the Interdisciplinary Center for Biotechnology Research at the University of Florida. Libraries were prepared using Illumina RNA-seq library preparation for low-input RNA and sequenced using the NovaSeq 6000 with paired-end libraries. More details on sequenced reads are available in Table 1.

### Differential gene expression analysis

We performed differential gene expression analyses on the *B. ovata* transcriptomic data we generated and on previously published transcriptomic data collected from the comb rows (35) and the aboral organs (76) of adult *M. leidyi*. These comparisons were used to investigate tissue-defining genes shared by the last common ancestor of *B. ovata* and *M. leidyi*. We aligned all transcript reads from *B. ovata* and *M. leidyi* to the *B. ovata* and *M. leidyi* (ML2.2) gene models, respectively, using Bowtie 2 (77) and estimated transcript abundance with RSEM (78) implemented in the Trinity (79) package script align_and_estimate_abundance.pl. We generated a principal component analysis (PCA) with the Perl-to-R script from the Trinity package with single-copy orthologs (see the next paragraph). We performed differential gene expression analyses comparing data collected from each type of tissue using limma + voom (80), executed through the Trinity script run_DE_analysis.pl.

After differential gene expression analysis of the tissue-specific data, we filtered results to include only single-copy orthologs to identify conserved genes. To identify single-copy orthologs, we inferred orthogroups using OrthoFinder and varied the MCL inflation parameter (-i) to 1.5, 5, 8 and 10. In addition to the one-to-one orthologs identified by OrthoFinder, we searched the phylogenetic trees generated by OrthoFinder for monophyletic pairs of *Mnemiopsis* and *Beroe* sequences.

We combined the single-copy orthologs from each OrthoFinder run (we found no conflicting results) using the custom script print_total-1_to_1s.pl. This produced a total of 5038 single-copy orthologs. We added 443 single-copy orthologs to this set by performing phylogenetic analyses on non-single-copy orthogroups. This was done by reconstructing gene trees from orthogroups containing four or more genes where the MCL inflation parameter was set to 1.5 (default) in OrthoFinder. We aligned genes in MAFFT v7.407 (81) and built maximum-likelihood trees with IQ-TREE using the model LG + G4 and a midpoint root. We designated genes as single-copy orthologs in cases where one *B. ovata* and *M. leidyi* gene formed a clade. To build alignments and gene trees, we used the custom script ogs_w_4plus_taxa_mafft_iqtree.pl (in our project GitHub repo). For each OrthoFinder run, we also identified genes that were not considered single-copy orthologs due to the presence of multiple isoforms (we discarded four genes that conflicted under various MCL inflation parameter settings). We identified a total of 1511 genes that could be considered single-copy orthologs using the custom script print_isoforms_blocking_one_to_ones.pl (in our project GitHub repo). We combined the single-copy orthologs identified from all the OrthoFinder runs, gene trees and orthogroups containing multiple isoforms, and removed genes that overlapped between the gene tree and isoform analysis using the custom script get_final_set.pl (in our project GitHub repo). In total, we identified 6348 single-copy orthologs.

After filtering the results from the differential gene expression analysis to only single-copy orthologs, we set criteria to detect statistically significant differences in gene expression. Thus, we considered the difference in expression profiles of single-copy orthologous genes with a false discovery rate of ≤0.001 and an absolute value of log_2_ fold change ≥2 in one species and a false discovery rate of ≤0.01 in the other species to be statistically significant differences in gene expression. We identified these using the script make_5488_dge_csv.pl (in our projectGitHub repo) and determined the function of genes with significant differences in gene expression by performing a BLASTP search against the human RefSeq database. In cases where there were no hits to the human RefSeq database, we performed a BLASTP search against the complete RefSeq database. We performed additional analyses including searching Pfam-A domains for genes of interest (see Supplementary Data).

## Results

### A high-quality complete genome for *B. ovata*

We generated 27 218 713 Illumina paired-end genomic DNA reads and 209 960 857 PacBio genomic DNA reads. The estimated genome size for the individual sequenced with Illumina was 94.3 Mb (Supplementary Figure S3). We assembled these data into 4989 scaffolds with 91 597 139 base pairs and an N50 of 233 527 (Table 2). BUSCO scores to assess the completeness of our assembly were 211 (82.75%) for complete core eukaryotic genes and 237 (92.94%) for complete and partial core eukaryotic genes out of the 255 total BUSCO core eukaryotic genes (Table 2). CEGMA scores to assess genomic assembly completeness were 227 (91.53%) for complete core eukaryotic genes and 241 (97.18%) for complete and partial core eukaryotic genes of the 248 total CEGMA core eukaryotic genes (Table 2). We predicted 13 762 protein-coding genes (Table 2). With isoforms included, we predicted a total of 18 664 transcript models. BUSCO scores to assess completeness of protein models were 230 (90.20%) for complete core eukaryotic genes and 244 (95.69%) for complete and partial core eukaryotic genes.

**Table 2.** Comparison of genome assembly and annotation summary statistics of *B. ovata* and other published ctenophore genomes

	***Beroe ovata***	***Mnemiopsis leidyi***	***Pleurobrachia bachei***	***Hormiphora californensis***
Sequence length (bp)	91 597 139	155 865 547	156 121 975	110 691 255
Sequences	4989	5100	21 979	45
N50 (bp)	233 527	187 314	20 628	8 537 259
GC content	41.97	37.49	37.56	43.13
bp in sequences ≥25 kb	81 735 191	143 875 185	70 888 134	110 434 894
BUSCO complete	82.75%	91.75%	86.14%	80.78%
BUSCO complete & part	92.94%	96.70%	94.39%	90.20%
CEGMA complete	91.53%	83.87%	77.02%	86.69%
CEGMA complete & part	97.18%	95.97%	93.95%	95.56%
Genes predicted	13 762	16 548	19 524	14 265
Interspersed repeats	10.00%	20.91%	13.58%	28.92%

bp: base pairs; kb: kilobases; complete & part denote scores for complete and partial core eukaryotic genes. The Eukaryota database used in the BUSCO analysis contained 255 core genes.

The *B. ovata* assembly surpassed previous non-chromosome-level ctenophore assemblies (i.e. *M. leidyi* and *P. bachei* assemblies) based on quality N50 metrics (Table 2). As expected, the chromosome-level *H. californensis* assembly had the largest N50 of the assembled ctenophore genomes (Table 2). The CEGMA scores were higher for the *B. ovata* assembly compared to all previous ctenophore assemblies, including the chromosome-level assembly of *H. californensis* (Table 2). The BUSCO scores of the *B. ovata* genome assembly were lower than those of the non-chromosome-level assemblies of *M. leidyi* and *P. bachei*, but surprisingly higher than those of the *H. californensis* assembly (Table 2). Our analysis of assembly repetitive content in *B. ovata* revealed a low proportion of repetitive elements, where 10% of the genome is composed of interspersed repeats. This was the lowest proportion of repetitive content of the published ctenophore genomes (Table 2). Repetitive elements did not scale with genome size in ctenophores (Table 2). However, estimates of repetitive elements are dependent on genome assembly quality, where chromosome-level assemblies will provide reasonably accurate estimates, while non-chromosomal assemblies are likely to underestimate repeats as they are often compacted or represented by gaps. Since the assembly of the *B. ovata* genome was based on Illumina short reads, the low estimation of repetitive elements may be an underestimation.

### Thirteen chromosomes in *B. ovata*

**Figure 2.**
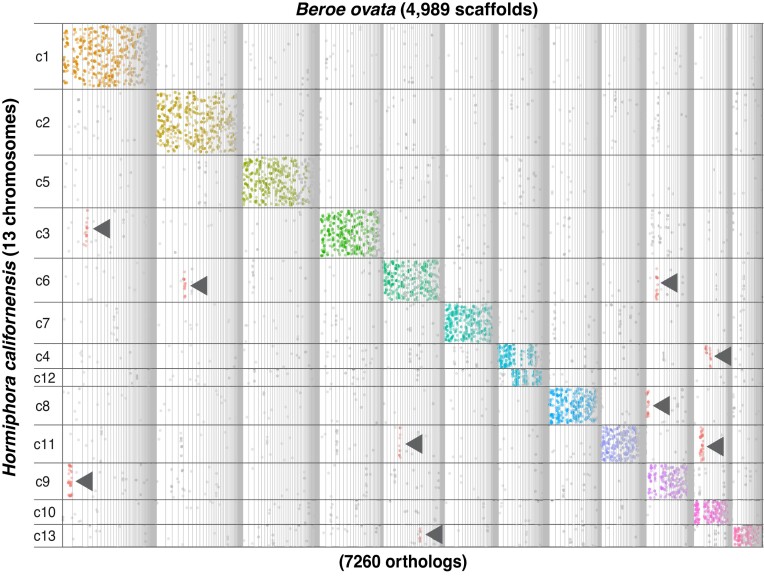
Oxford dot plot of macrosynteny between *B. ovata* and *H. californensis*. The coordinates of 7260 orthologs (based on reciprocal best BLAST hits) are plotted on *H. californensis* chromosomes and *B. ovata* scaffolds (full list of genes and corresponding scaffolds is available on the project GitHub repo). Each dot represents an ortholog. Arrowheads indicate potential translocations.

We performed an analysis of macrosynteny between *B. ovata* and *H. californensis*, where we identified conserved genes on the scaffolds of *B. ovata* and on the chromosomes of *H. californensis* without considering gene order. Our analyses suggest that *B. ovata*, like *H. californensis*, has 13 chromosomes since most of the *B. ovata* scaffolds map to mutually exclusive *H. californensis* chromosomes (Figure 2). The results also revealed a number of putative translocations where a linkage group has been dispersed across multiple *H. californensis* chromosomes (arrowheads in Figure 2). While a few translocations are not unexpected, it is possible that these could represent misassemblies. Nevertheless, this analysis largely corroborates the extensive scaffolding (>50 000 joins) that we applied to generate the assembly and provide the highest resolution to date regarding the number of chromosomes possessed by *B. ovata*.

### Genes are conserved in the *B. ovata* TGF-β pathway

**Figure 3.**
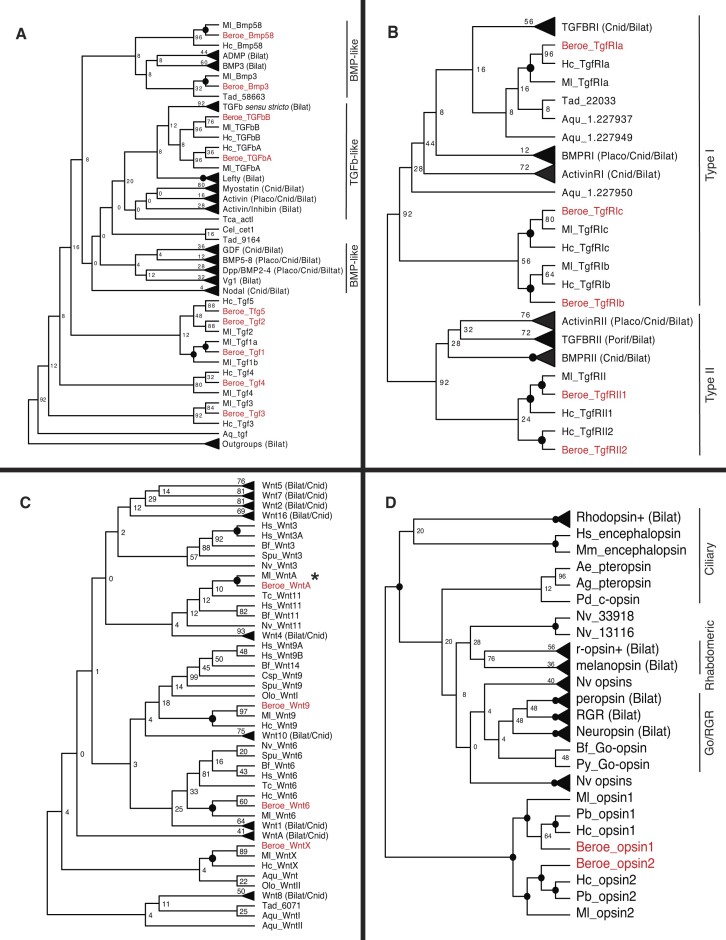
Maximum-likelihood gene family phylogenies. Subset of maximum-likelihood phylogenies chosen according to likelihood scores out of the full set of trees estimated under a range of algorithms and parameter settings (see the ‘Materials and methods’ section). Circles at nodes represent 100% bootstrap support. Triangles at tips represent collapsed clades that includes sequences from the lineages indicated in parentheses. Information associated with each gene is provided in Supplementary Table S4. (**A**) Phylogeny for TGF-β ligands produced using a maximum parsimony starting tree in RAxML. (**B**) Phylogeny for TGF-β receptors produced from IQ-TREE with default settings. (**C**) Phylogeny for Wnt ligands produced using random starting trees in RAxML. WntA for *H. californensis* was not included in this analysis because it was not annotated in the current version of the *H. californensis* genome annotations. (**D**) Phylogeny for opsins produced from IQ-TREE with default settings. Labels abbreviated with Ml, Pb and Hc are genes from *M. leidyi*, *P. bachei* and *H. californensis*, respectively.

We built upon the maximum-likelihood phylogenetic analyses of TGF-β ligands and receptors of Pang *et al.* (69) by adding data from *B. ovata* and *H. californensis*. In the previous study, nine TGF-β ligands and four receptors were found in *M. leidyi*. We identified nine TGF-β ligands and five receptors in *B. ovata* and found six TGF-β ligands and five receptors in *H. californensis* (Figure 3A and B).

As in (69), there were two ctenophore TGF-β-like families, TGFbA and TGFbB. In the previous study, *M. leidyi* TGFbA was most closely related to the TGF-β *sensu stricto* family, while *M. leidyi* TGFbB was most closely related to the Lefty family. In our analysis, ctenophore TGFbA and TGFbB sequences were most closely related to each other, with low support as sister to the TGF-β *sensu stricto* family (BS = 12; Figure 3A). The remaining ctenophore TGF-β ligands labeled Tgf1, Tgf2, Tgf3, Tgf4 and Tgf5 grouped as sister to the TGF-β-like and BMP-like families (Figure 3A), consistent with Pang *et al.* (69). We found that *H. californensis* lost Bmp3, and that either *H. californensis* lost the ctenophore-specific Tgf1 and Tgf2 ligands or these arose after *H. californensis* diverged from *M. leidyi* and *B. ovata* (Figure 3A). Pang *et al.* (69) identified a Tgf1 duplication in *M. leidyi*. From our inclusion of additional ctenophore data, we found that the duplication of Tgf1 in *M. leidyi* identified in (69) likely preceded the split of *Mnemiopsis* and *Beroe* (Figure 3A; see ctenophore phylogeny in Figure 1B). We also identified a loss of the Tgf5 ligand in *M. leidyi*, which is present in *B. ovata* and *H. californensis* (Figure 3A).

Ctenophore type II TGF-β receptors formed a clade within a larger well-supported type II receptor clade (BS = 92; Figure 3B). Likewise, ctenophore type I TgfRIa receptors formed a clade within a larger clade of type I receptors, albeit with weak support (BS = 16; Figure 3B). The ctenophore TgfRIb and TgfRIc receptors identified in (69) grouped within the clade of type I receptors (BS = 92; Figure 3B). We identified two type II receptors in *B. ovata* and *H. californensis* (i.e. TgfRII1 and TgfRII2), whereas only a single type II receptor was identified in *M. leidyi* (i.e. TgfRII) (69), suggesting that a type II receptor was lost in *M. leidyi* (Figure 3B; see ctenophore phylogeny in Figure 1B). We also identified the TgfRIa, TgfRIb and TgfRIc type I receptors in *H. californensis* and *B. ovata* that were previously characterized in *M. leidyi* (69).

### Genes are conserved in the *B. ovata* Wnt pathway

We added data from *B. ovata* and *H. californensis* into the alignment of Wnt ligands from Pang *et al.* (70) and performed a maximum-likelihood phylogenetic analysis. The previous study identified four Wnt ligands in *M. leidyi* (70). We identified four Wnt ligands in *B. ovata* and three Wnt ligands in *H. californensis* (Figure 3C). Pang *et al.* (70) identified Wnt6, Wnt9, WntA and WntX in *M. leidyi*. In that study, Wnt6, Wnt9 and WntA grouped with cnidarian and bilaterian Wnt families with moderately strong support (BS ≥ 75), but WntX did not group with any recognized gene families. Our results showed weak support uniting ctenophore Wnt6, Wnt9 and WntA gene families with those of Cnidaria and Bilateria (BS < 50; Figure 3C) and weak support for grouping WntX genes with WntI and WntII genes identified in sponges (BS = 4; Figure 3C).

We found Wnt6, Wnt9 and WntX ligands in all ctenophore species included in this analysis (i.e. *B. ovata, H. californensis* and *M. leidyi*). Ctenophore Wnt6 genes grouped together in a clade with strong support (BS = 100; Figure 3C) and were weakly supported in a larger Wnt6 clade including genes from cnidarians and bilaterians (BS = 33; Figure 3C). While ctenophore Wnt9 ligands grouped together in a clade with strong support (BS = 100; Figure 3C), they were weakly supported in a larger clade that included Wnt9 and Wnt14 sequences from Bilateria and a WntI sequence from a sponge (BS = 18; Figure 3C). Similarly, ctenophore WntX genes grouped together in a clade with strong support (BS = 100; Figure 3C) and were weakly supported in a larger clade that contained one placozoan, several sponge, cnidarian and bilaterian genes including Wnt8 (BS < 50; Figure 3C).

We found WntA genes in *B. ovata* and *M. leidyi*. These genes grouped together in a clade with strong support (BS = 100; Figure 3C), but grouped within a larger clade of cnidarian and bilaterian Wnt11 genes with weak support (BS = 12; Figure 3C). We did not find a WntA gene in the current version of the *H. californensis* genome annotations, but after a reviewer noted the missing gene, we confirmed a reciprocal best BLAST hit to an unannotated WntA gene in the *H. californensis* genome assembly. Thus, while a WntA sequence for *H. californensis* was not included in our phylogeny, WntA is conserved across the ctenophore lineages.

### Opsins are conserved across ctenophores

Given that *Beroe* ctenophores use a raptorial predation strategy unique among other ctenophores, we tested for differences in the number and types of opsin genes present in the *B. ovata* genome compared to other ctenophores. We identified two opsins in *B. ovata* and two opsins in *H. californensis*. Schnitzler *et al.* (71) identified two opsins in *Pleurobrachia pileus* and three opsins in *M. leidyi* based on conserved EF-hand domain residues (i.e. found in a larger gene family of calcium-binding proteins, including opsins). However, in our preliminary phylogenetic analyses that included a large number of non-opsin G-protein-coupled receptors, *Mnemiopsis* opsin3 did not group with true opsins (Supplementary Figure S1). Rather, *Mnemiopsis* opsin3 occurred within a large clade of non-opsin G-protein-coupled receptors, the vast majority of which lacked the lysine residue from helix VII, which is critical for Schiff base binding of the chromophore and is a defining feature of functional opsins. As such, we do not consider *Mnemiopsis* opsin3 a *sensu stricto* opsin and removed it from our final phylogenetic analysis.

Our results show that opsin1 and opsin2 are conserved across ctenophores (Figure 3D). Furthermore, our results suggest that ctenophore opsins are more closely related to ciliary opsins than to Go-coupled plus retinochrome, retinal G-protein-coupled receptor (Go/RGR) and rhabdomeric opsins (Figure 3D). This is somewhat different from Schnitzler *et al.* (71), where ctenophore opsins were more closely related to both ciliary and Go/RGR opsins than to rhabdomeric opsins, although support for these deeper relationships was low in both studies (BS < 50 in both studies; Figure 3D).

### *Beroe ovata* lacks a functional chitinase gene and many tentacle genes

We hypothesized that the derived ctenophorivorous feeding behavior of *Beroe* may have led to the loss of chitinase, the enzyme used for digesting the chitin, which is found in many animals, including the exoskeleton of crustaceans. We tested this hypothesis by scanning both the *B. ovata* genome assembly and gene models. We did not find any evidence of a chitinase with a conserved catalytic motif (FDG(X)DXDXE) (82) in the *B. ovata* genome (see Supplementary Data). In contrast, we recovered three chitinase genes each from the genomes of *M. leidyi* (ML368913a, ML07445a, ML03134a) and *H. californensis* (Hcv1.av93.c5.g1106.i1, Hcv1.av93.c11.g13.i1, Hcv1.av93.c13.g295.i1), all of which had the catalytic motif. We also recovered one chitinase-like protein from *B. ovata* (Bova1_4.0228.g14.t1), *M. leidyi* (ML003271a) and *H. californensis* (Hcv1.av93.c11.g213.i1), each of which has a significant Glyco-hydro_18 domain but lacks an active catalytic site and therefore likely lacks the enzymatic activity.

Babonis *et al.* (35) showed that *Beroe* lost 354 genes that were present in most other ctenophore lineages. In *M. leidyi*, these 354 genes were significantly enriched in cell types associated with tentacles and tended to be effector genes rather than transcription factors. The *Beroe* absences were based primarily on three transcriptomes (*B. ovata*, *B. forskalii* and *B. abyssicola*) as well as a very early draft genome of *B. ovata*. We looked for these 354 genes in our *B. ovata*–*M. leidyi* OrthoFinder analyses and found no evidence for these genes in the current *B. ovata* genome, thus corroborating the previous results.

### The identification of a core set of comb plate and aboral organ genes

**Figure 4.**
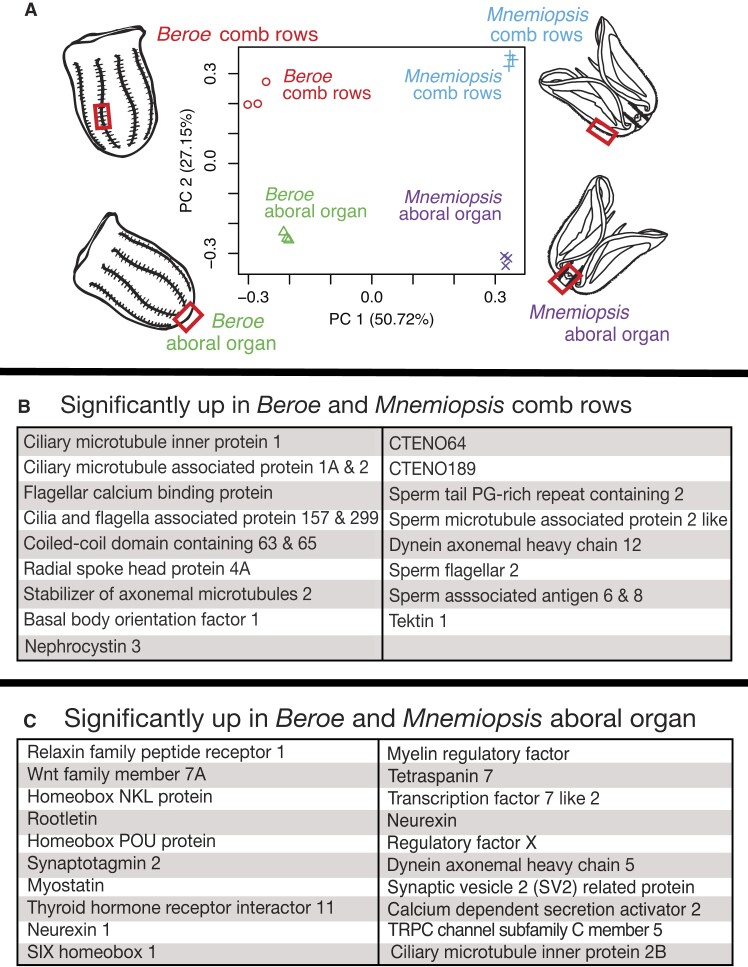
Conserved differential gene expression in the ctenophore comb rows and aboral organ. Genes were annotated by performing BLASTP searches against the human RefSeq or complete RefSeq databases. (**A**) PCA of transcriptomic data collected from the comb rows and aboral organs of *B. ovata* and *M. leidyi*. (**B**) Conserved genes with significantly higher expression in *B. ovata* and *M. leidyi* comb rows relative to the aboral organ. (**C**) Conserved genes with significantly higher expression in the aboral organ relative to the comb rows.

To detect tissue-specific genes present in the last common ancestor of *B. ovata* and *M. leidyi*, we performed a PCA and differential gene expression analyses on single-copy orthologs that were identified with OrthoFinder. PC1 accounted for 50.72% of the total variance where samples were united by species, while PC2 accounted for 27.15% of the variance where samples were united by tissues (Figure 4A). We compared gene expression profiles of *B. ovata* comb rows with those of *B. ovata* aboral organs as well as *M. leidyi* comb rows and *M. leidyi* aboral organs. In terms of overlap between species comparisons, we identified a total of 55 one-to-one orthologs expressed significantly higher in comb rows relative to aboral organs and 45 genes expressed significantly higher in aboral organs relative to comb rows (Supplementary Tables S2 and S3).

We expected that the comb rows would be defined by the expression of genes related to ciliary function, while the aboral organs would be defined by the expression of a wide range of genes due to the high variation of cell types (e.g. sensory cells, cilia, lithocytes, etc.) present in these tissues (83). To determine the putative function of highly expressed tissue-specific genes, we performed a BLASTP search against the human RefSeq database. For any genes lacking hits to human RefSeq, we followed up with a BLASTP search against the complete RefSeq database. Many genes expressed significantly higher in the comb rows relative to the aboral organ had top hits to genes associated with cilia and/or sperm, including ciliary microtubule inner protein 1 (Bova1_5.0211.g20), ciliary microtubule associated protein 1A (Bova1_5.0092.g21), ciliary microtubule associated protein 2 (Bova1_5.0082.g21), flagellar calcium-binding protein (Bova1_5.0074.g42), cilia and flagella associated protein 157 (Bova1_5.0039.g17), cilia and flagella associated protein 299 (Bova1_5.0025.g66), coiled-coil domain containing 63 (Bova1_5.0022.g12), coiled-coil domain containing 65 (Bova1_5.0036.g27), radial spoke head component 4A (Bova1_5.0019.g96), stabilizer of axonemal microtubules 2 (Bova1_5.0206.g16), basal body orientation factor 1 (Bova1_5.0002.g47), sperm tail PG-rich repeat containing 2 (Bova1_5.0195.g11), sperm microtubule associated protein 2 like (Bova1_5.0003.g46), dynein axonemal heavy chain 12 (Bova1_5.0009.g74), sperm flagellar 2 (Bova1_5.0042.g60), sperm associated antigen 8 (Bova1_5.0097.g35), sperm associated antigen 6 (Bova1_5.0097.g35), tektin 1 (Bova1_5.0020.g123), nephrocystin 3 (Bova1_5.0086.g15) and two recently described ctenophore-specific comb row genes [i.e. CTENO64 (84) and CTENO189 (85)] (Figure 4B and Supplementary Table S2).

A diverse set of genes were highly expressed in the aboral organ relative to the comb rows. Of the 45 genes expressed significantly higher in the aboral organ relative to the comb rows, several of these had top hits to genes associated with the nervous system, development and cell proliferation, cilia and signal transduction. Genes with top hits that were associated with the nervous system included synaptotagmin 2 (Bova1_5.0168.g20), neurexin 1 (Bova1_5.0050.g30), thyroid hormone receptor interactor 11 (Bova1_5.0175.g10), myelin regulatory factor (Bova1_5.0128.g29), synaptic vesicle 2 related protein (Bova1_5.0389.g7), transient receptor potential cation channel subfamily C member 5 (Bova1_5.0059.g26) and calcium dependent secretion activator 2 (Bova1_5.0252.g5) (Figure 4C and Supplementary Table S3). Genes involved in development and cell proliferation included Wnt family member 7A (Bova1_5.0064.g51), homeobox NKL protein (Bova1_5.0391.g4), homeobox POU protein (Bova1_5.0024.g99), SIX homeobox 1 (Bova1_5.0066.g6), transcription factor 7 like 2 (Bova1_5.0068.g8), myostatin (Bova1_5.0002.g73) and regulatory factor X1 (Bova1_5.0015.g66) (Figure 4C and Supplementary Table S3). Genes associated with cilia that were highly expressed in the aboral organ relative to the comb rows included rootletin (Bova1_5.0216.g7), dynein axonemal heavy chain 5 and ciliary microtubule inner protein 2B (Figure 4C and Supplementary Table S3). Finally, genes involved in signal transduction were those with top hits to relaxin family peptide receptor 1 (Bova1_5.0057.g17) and tetraspanin 7 (Bova1_5.0075.g46).

Eleven of the highly expressed tissue-specific genes (i.e. six genes expressed significantly higher in the comb rows relative to the aboral organ and five genes expressed significantly higher in the aboral organ relative to the comb rows) could not be characterized as they had no hits to homologous genes from the BLASTP search against the human and complete RefSeq databases (Supplementary Tables S2 and S3), suggesting that these may be highly derived or ctenophore-specific genes.

### BovaDB

We created the *B. ovata* Database (BovaDB) to make the *B. ovata* genome assembly, annotation, gene models, protein models, preliminary mitochondrial genome assembly and annotation, and tissue-specific transcriptomic data conveniently accessible. BovaDB provides two BLAST interfaces, including NCBI BLAST with a graphical view (86) and ViroBLAST (87), which provides the ability to download sequences that correspond to BLAST hits. The BLAST databases include the *B. ovata* genome assembly, gene models, protein models and transcriptomic data. This database is available at http://ryanlab.whitney.ufl.edu/bovadb.

BovaDB also includes gene pages in which annotation features for each gene can be viewed, including scaffolds, data type, exonic genomic coordinates and corresponding transcripts (Supplementary Figure S4). Protein and nucleotide sequences for coding regions are provided. Each gene page includes reports of precomputed BLASTP hits for the *B. ovata* protein models searched against protein sequences in the human RefSeq database, the UniProt database, *M. leidyi* protein models and *H. californensis* protein models. Reported BLASTP hits to proteins in each database are hyperlinked by accession numbers to their entries in RefSeq, UniProt and the *Mnemiopsis* Genome Project Portal. *E*-values are provided for each BLASTP hit and only hits to proteins with an *E*-value <0.001 were included.

For each gene page, we also provide protein domains detected from the Pfam-A database in the *B. ovata* protein models with hmmscan from HMMER v.3.3 (88). Detected Pfam-A domains are hyperlinked to their corresponding entries on the Pfam website (89). *E*-values and alignment start and end positions from hmmscan searches are included for reference. Additionally, each gene page incorporates gene expression profiles of transcriptomic data collected from the comb rows and the aboral organ of *B. ovata*. We use box plots to visualize mapped reads in transcripts per million for all replicates (i.e. three) that were collected from the comb rows and aboral organ (Supplementary Figure S4). Gene expression profiles were produced using ggplot2 (90) in R (91).

## Discussion

### *Beroe ovata* genome is compact

Here, we present the sequence, assembly and annotation of the *B. ovata* genome. We have generated a high-quality *B. ovata* genomic assembly produced by scaffolding Illumina short-read contigs with artificial mate pairs generated from assemblies of PacBio long reads. Notably, the *B. ovata* assembly is the most compact of the published ctenophore genomes (91 Mb) with the smallest number of predicted genes (13 762 genes; Table 2). These data suggest that many genes were lost in *Beroe* after divergence from the last common ancestor of *Beroe* and *Mnemiopsis* (Figure 1). While *B. ovata* has the smallest number of predicted genes among ctenophores, we observed several early developmental and sensory-related genes that remained conserved, whereas many effector genes that were associated with structures or behaviors that were lost in the *Beroe* lineage were absent. These observations are consistent with previous findings from (35).

### Gene families conserved in *B. ovata*

Upon sequencing of the *M. leidyi* genome more than a decade ago, a number of evolutionary studies of gene families that incorporated these new data were published (69–71,92). Despite the increasing availability of genome data from additional ctenophore species, these studies have not been revisited, and apart from gene synteny (14), very few comparative genomic analyses have been performed across ctenophore species. Results from our analyses revealed that genes are conserved in the TGF-β and Wnt pathways in *B. ovata* (Figure 3). Beroids are morphologically unique among ctenophores as they lack a tentacle-bearing life stage and develop macrociliary teeth for predatory feeding very early in development. A previous study investigating the loss of tentacles in the genus *Beroe* identified over 300 genes related to colloblasts and tentacles that were missing from this lineage (35). Very few of these missing genes were developmental regulatory genes. This suggests that upstream regulators of tissue morphogenesis may be highly conserved despite variation in the adult body plan. Indeed, in *B. ovata*, we show that many genes involved in early development were conserved.

Our phylogenetic analyses showed that a core set of opsins (opsin1 and opsin2) were present in the last common ancestor of ctenophores (Figure 3D). Schnitzler *et al.* (71) identified an additional *M. leidyi* G-protein-coupled receptor that they named *Mnemiopsis* opsin3 that included a diagnostic lysine residue. Analyses by Schnitzler *et al.* (71) showed that *Mnemiopsis* opsin3 did not group with other ctenophore opsins and was placed as sister to the rest of the opsins included in their analyses with low support and certainty. On the other hand, *Mnemiopsis* opsin3 was placed in a clade that included *Mnemiopsis* opsin1 and opsin2 along with cnidarian and bilaterian opsins in (93). Our phylogenetic analyses, which included a greatly expanded set of non-opsin genes with EF-hand domains (see the ‘Materials and methods’ section and Supplementary Data), placed *Mnemiopsis* opsin3 in a clade with non-opsins (Supplementary Figure S1). Yet, *Mnemiopsis* opsin3 has a conserved lysine residue at helix VII, an important site for Schiff base binding that is highly conserved in opsins (71). Schnitzler *et al.*(71) also identified a conserved glutamic acid at site 181 in *Mnemiopsis* opsin3, a potential site for the Schiff base counterion that stabilizes protonation of the Schiff base, as well as opsins without a bound chromophore (94). These portions of the sequence that resemble defining opsin features [indeed the criteria for inclusion in (71)] and the phylogenetic position of *Mnemiopsis* opsin3 suggest that *Mnemiopsis* opsin3 may represent a rare case of convergent sequence evolution in a photoreceptor gene.

### Gene loss is associated with change in diet and regenerative ability in *B. ovata*

Most animals can synthesize chitin (95), but chitin synthase is not present in the *Mnemiopsis* genome (96), and based on phylogenetic analyses (97), as well as biophysical characteristics, ctenophores are not thought to be able to produce chitin (98). However, the use of highly sensitive proteomic analyses is needed to confirm the absence of chitin in ctenophores. There was once debate regarding the diet of beroids—whether they are exclusive predators of other ctenophores or whether they still ingest crustaceans when possible (27). Behavioral observations show that beroids egest indirectly ingested chitinous organisms when feeding on ctenophore prey that include these chitinous organisms as undigested gut contents (99). We show that *B. ovata* lacks a recognizable chitinase and therefore, like the behavioral observations, supports the idea that *Beroe* cannot digest chitin. This contrasts the condition of *M. leidyi* and *H. californensis*, species known to digest chitinous organisms (100), which have three functional chitinase genes with active catalytic sites.

Intriguingly, a recent study of the sea anemone *Nematostella vectensis* identified a chitinase gene as one of the top differentially expressed genes during regeneration (101). This suggests that in addition to a dietary function, chitinase may play a role in wound healing and/or regeneration. It is interesting that *M. leidyi* and other ctenophores have proper chitinase genes and are able to undergo whole-body regeneration, while *B. ovata* lacks a functional chitinase and is unable to undergo whole-body regeneration. In light of these observations, we hypothesize that the loss of chitinase contributes to loss of regenerative abilities within *Beroe*.

### A core set of comb plate and aboral organ genes in the last common ancestor of *B. ovata* and *M. leidyi*

Ctenophores possess eight comb rows that line their bodies and are composed of thousands of cilia that beat to create movement to swim toward prey or escape from predators. These movements are controlled by the aboral organ, a ctenophore-specific structure composed of a wide range of cell types, including epithelial cells (102), lithocytes (103), nerve cells (104,105), cilia (106) and photoreceptors (107). Our data provide a strong inference for the core set of genes associated with the comb rows and aboral organ in the ancestor of *M. leidyi* and *B. ovata*.

Our results showed that many of the core comb plate genes were homologous to those involved in cilia formation, assembly and function, as well as four genes associated with sperm (Figure 4B and Supplementary Table S2). The highly expressed genes associated with sperm likely resulted because ctenophores are hermaphroditic and possess male and female gametes in the meridional canals located beneath the comb rows (108). Therefore, sperm cells were most likely sequenced inadvertently with our comb row tissue. Included as highly expressed genes in the comb rows relative to the aboral organ were two previously described ctenophore-specific genes, CTENO64 and CTENO189 (84,85) (Figure 4A). CTENO64 and CENO189 are primarily expressed in the compartmenting lamella, a unique ctenophore structure that connects adjacent cilia in the comb plates and ensures proper orientation of cilia for coordinated beating (84,85). Many of the remaining genes were those with general functions or involved in metabolism, cell cycling and cell signaling (Supplementary Table S3).

For differential gene expression in the aboral organs relative to the comb rows, we found 45 conserved genes expressed significantly higher in both *B. ovata* and *M. leidyi*. We observed greater diversity in terms of function in the core aboral organ genes compared to the core comb row genes. This is not entirely unexpected, as this diversity likely reflects the variability of cell types present in the aboral organs relative to that of comb rows, which tend to be dominated by comb plate cilia. The aboral organ is described as a neurosensory complex with a nerve net and integrated neurites (8,109), where our results reveal several highly expressed genes involved in this neural system that will serve as key genes for further investigations on the evolution of the animal nervous system. Further, given that one of the core processes occurring in the ctenophore aboral organs is the continuous development of lithocytes, we hypothesize that the developmental genes (e.g. homeobox genes) that are highly expressed in the aboral organs of both *Beroe* and *Mnemiopsis* are involved in lithocyte specification. Thus, a key next step to better understanding the ctenophore aboral organ will be to investigate these 45 core aboral organ genes.

## Conclusion

Our analyses include an overall comparison of genome size and gene number, detailed phylogenetic analyses of key gene families and cross-species comparisons of gene expression profiles of tissues. The *B. ovata* genome is substantially smaller compared to previously sequenced ctenophore genomes. Our phylogenetic analyses show that this lineage retained conserved early developmental gene families and genes related to the sensory system. Our gene content analyses identify the loss of downstream effector genes associated with ancestral characteristics that were lost in the *Beroe* lineage (e.g. chitin digestion and tentacle cell types). Lastly, our comparative gene expression analyses provide a set of core candidate genes underlying key structures defining the ctenophore body plan. Together, these analyses provide a first glance into the evolutionary dynamics of the *Beroe* lineage.

To date, genomic comparisons involving ctenophores have been dominated by comparisons with non-ctenophore species rather than intra-Ctenophora comparisons. We anticipate that over the next decade, genomic comparisons between ctenophore species will become more common and greatly expand our understanding of genomic evolution within Ctenophora. We also predict that, like comparisons of ctenophores with other animals, comparisons within Ctenophora will lead to novel biological insight and unexpected findings.

## Supplementary Material

lqae072_Supplemental_File

## Data Availability

All genomic and transcriptomic data have been made available in the BovaDB (http://ryanlab.whitney.ufl.edu/bovadb/). Genomic data collected from Illumina sequencing have been deposited in the European Nucleotide Archive (ENA) under the study number PRJEB23672 with accession numbers ERR2206326 and ERR2206327. Genomic data collected from the PacBio sequencing were submitted to ENA using the same study number and are found under accession numbers ERR9814540, ERR9829220, ERR9829405, ERR12051222 and ERR9829408–ERR9829411. The genomic assembly was deposited under the accession number GCA_900239995. Transcriptomic data used for gene annotations have been made available in ENA under study number PRJEB23650 and under accession numbers ERR2205101–ERR2205121. Tissue-specific transcriptomic data were also deposited to ENA under study number PRJEB55009 with accession numbers ERR9993350, ERR9993422, ERR10006118, ERR10009774, ERR10009850 and ERR10016030. BovaDB is available at http://ryanlab.whitney.ufl.edu/bovadb/.
