# Supplementary material for: Morphological and dietary changes encoded in the genome of Beroe ovata, a ctenophore-eating ctenophore
Source: NAR Genom Bioinform. 2024 Jun 18;6(2):lqae072. doi: 10.1093/nargab/lqae072 (PMC11184263; doi:10.1093/nargab/lqae072)
Supplement: lqae072_Supplemental_File [file lqae072_supplemental_file.pdf]

Supplementary Materials for

Morphological and dietary changes encoded in the genome of *Beroe ovata*, a ctenophore-eating ctenophore

Alexandra M. Vargas[1,2]†, Melissa B. DeBiasse[1,3]†, Lana L. Dykes[1], Allison Edgar[1,4], T. Danielle Hayes[1], Daniel J. Groso[1,5,6], Leslie S. Babonis[7], Mark Q. Martindale[1,8], Joseph F. Ryan[1,8]

This pdf includes:

Supplementary Methods
Supplementary Analyses
Figures S1-S4
Table S1-S4

Supplementary Methods

Transcriptome sequencing and assembly

We spawned adult *Beroe ovata* individuals in the lab and collected embryos at 20 hours post-fertilization. We extracted RNA from pooled embryos. Libraries were prepared using the Ambion PolyA kit, heat fragmentation, and the NEB Next Ultra kit. Sequencing was performed in both directions on the Illumina HiSeq 2500 at the University of Texas Austin Genomic Sequencing and Analysis Facility.

For the 20-hour time point, we trimmed adapters using the paired-end RNA-Seq reads using Trimmomatic. We assembled a transcriptome in Trinity v2.4.0 (1). We used the align_and_estimate_abundance.pl script in the Trinity package to identify the isoform with the most aligned reads and created a new assembly with these isoforms using the script rsemgetbestseqs.py (bitbucket.org/wrf/sequences/src). We translated the nucleotide transcriptome sequences into amino acid sequences in TransDecoder v5.0.2 (github.com/TransDecoder). We set the –m flag to 50 and used the results from BLAST (2) and hmmscan (3) searches to inform the final TransDecoder prediction step. This assembly was used to assess the gene predictions described in the main manuscript. Raw reads from developmental stages have been uploaded to the European Nucleotide Archive under project number PRJEB23650.

Mitochondrial genome assembly and annotation

We identified scaffolds originating from mitochondrial sequence data in a preliminary *B. ovat*a genome assembly using a BLASTN search against the *M. leidyi* mitochondrial genome assembly (4). We identified two scaffolds with significant hits to the *M. leidyi* mitochondrial genome and removed these from the *B. ovata* genome assembly. We assembled the mitochondrial genome by collapsing and consolidating overlap between the two scaffolds. We then used MITOS v2.1.7 (5) to generate a preliminary annotation for the assembly (commands available on our GitHub repo). Our preliminary assembly of the mitochondrial genome have been made available on the *Beroe ovata* DataBase (http://ryanlab.whitney.ufl.edu/bovadb).

Supplementary Analyses

Commands, alignments, and phylogenetic trees have been made available in the project GitHub repo: https://github.com/josephryan/Beroe_genome

Expanded phylogenetic analysis of opsins

Since *Mnemiopsis* opsin3 grouped in a clade of ctenophore non-opsins, we performed a phylogenetic analysis of non-opsin genes with EF-hand domains from *Mnemiopsis leidyi*, several cnidarians, and bilaterians. To perform this analysis, we downloaded protein models from *M. leidyi* and *Hydra vulgaris* from the *Mnemiopsis* and *Hydra* Genome Portals, from *Nematostella vectensis, Capitella teleta,* and *Drosophila melanogaster* from EnsemblMetazoa*,* and from *Homo sapiens* from NCBI (links to all the sources of data are on our GitHub repo). We used the script hmm2align.pl (6) to identify protein models containing EF-hand domains from *M. leidyi, Nematostella vectensis, Hydra vulgaris, Capitella teleta, Drosophila melanogaster,* and *Homo sapiens*. We performed a maximum-likelihood analysis of concatenated EF-hand domain-containing protein models using the multicore version of IQ-TREE v1.6.12 (7) with the LG model. Our results placed *Mnemiopsis* opsin3 in a clade of non-opsin G-protein-coupled receptors (Figure S2), consistent with our preliminary phylogenetic analysis (Figure S1).

Investigation of Beroe ovata chitinase

Since the identification of chitinase and chitinase-like genes in the *B. ovata* genome was dependent upon the quality of our genome annotation, we performed an additional analysis to confirm the lack of chitinase proteins observed. We aligned the ctenophore chitinase proteins we identified in *M. leidyi* (ML368913a, ML07445a, ML03134a) and in *Hormiphora californensis* (Hcv1.av93.c5.g1106.i1, Hcv1.av93.c11.g13.i1, Hcv1.av93.c13.g295.i1) to the *B. ovata* genome using miniprot (8). Only one ctenophore chitinase, ML368913a from *Mnemiopsis*, aligned to a *Beroe* assembly contig (Bova1_5.1697), albeit with a mapping quality score of 0 (out of 255) and an alignment score of 89. When we performed a BLASTX search of Bova1_5.1697 back to the *Mnemiopsis* protein models, the best hit was to a non-chitinase gene (ML095325a) with a 30.84% identity and an E-value of 1.4e-05 and the second-best hit was to the *Mnemiopsis* chitinase (ML368913a) with a 32.32% identity and an E-value of 1.37e-03. Further, our search for Pfam-A domains in Bova1_5.1697 produced no hits. We also conducted a regular expression search against a translated version of the genome for evidence of a conserved catalytic site characteristic of chitinase (FDG(X)DXDXE) and found that the surrounding sequences of the five hits either had no BLAST hits or hit non-chitinase genes in Uniprot (see fdgxdxdxe_in_genome.pl script in GitHub repo). Thus, we did not find evidence of a functional chitinase in the *B. ovata* genome.

Classifications of highly expressed developmental genes in aboral organs relative to comb rows

We identified 45 genes expressed significantly higher in the aboral organs relative to the comb rows and performed a BLASTP search against the human RefSeq database to classify these genes. For genes lacking a significant BLASTP hit to the human RefSeq database, we followed up by performing a BLASTP search against the complete RefSeq database. We classified 7 of the 45 genes with roles in development and cell proliferation, where Bova1_5.0391.g4 (ML02215a) and Bova1_5.0024.g99 (ML18891a) had significant hits to homeobox proteins (Table S3). We performed additional analyses on these genes (outlined below) to confirm homeobox classifications using the *M. leidyi* homeobox data and classifications produced by Ryan et al. (2010) (9).

To confirm the homeobox classifications of ML02215a and ML18891a in our study, we searched for these genes in the Ryan et al. (2010) (9) homeodomain superfamily alignment. We found that ML02215a was the same protein that was referred to as MlANTP37 in Ryan et al. (2010) (9) and was characterized as belonging to the NKL subclass of ANTP-class homeoboxes in that study. ML18891a was not included in the Ryan et al. (2010)(8) homeodomain superfamily alignment. As such, we performed a phylogenetic analysis of ML18891a concatenated to the Ryan et al. (2010)(8) homeodomain superfamily alignment. We ran a maximum-likelihood analysis in IQ-TREE using the LG+G4 model. Additionally, we searched for Pfam-A domains in ML18891a using the *Mnemiopsis* Genome Portal GeneWiki page. The results from our phylogenetic analysis supported the classification of ML18891a as a homeobox gene, where ML18891a was placed as sister to all other POU genes (the resulting tree file is available in the project GitHub repo). Further, our analysis of Pfam-A domains in ML18891a resulted in hits to a POU domain (E-value = 2.8e-28) and a homeodomain (E-value = 2.0e-20).

Supplementary Figures

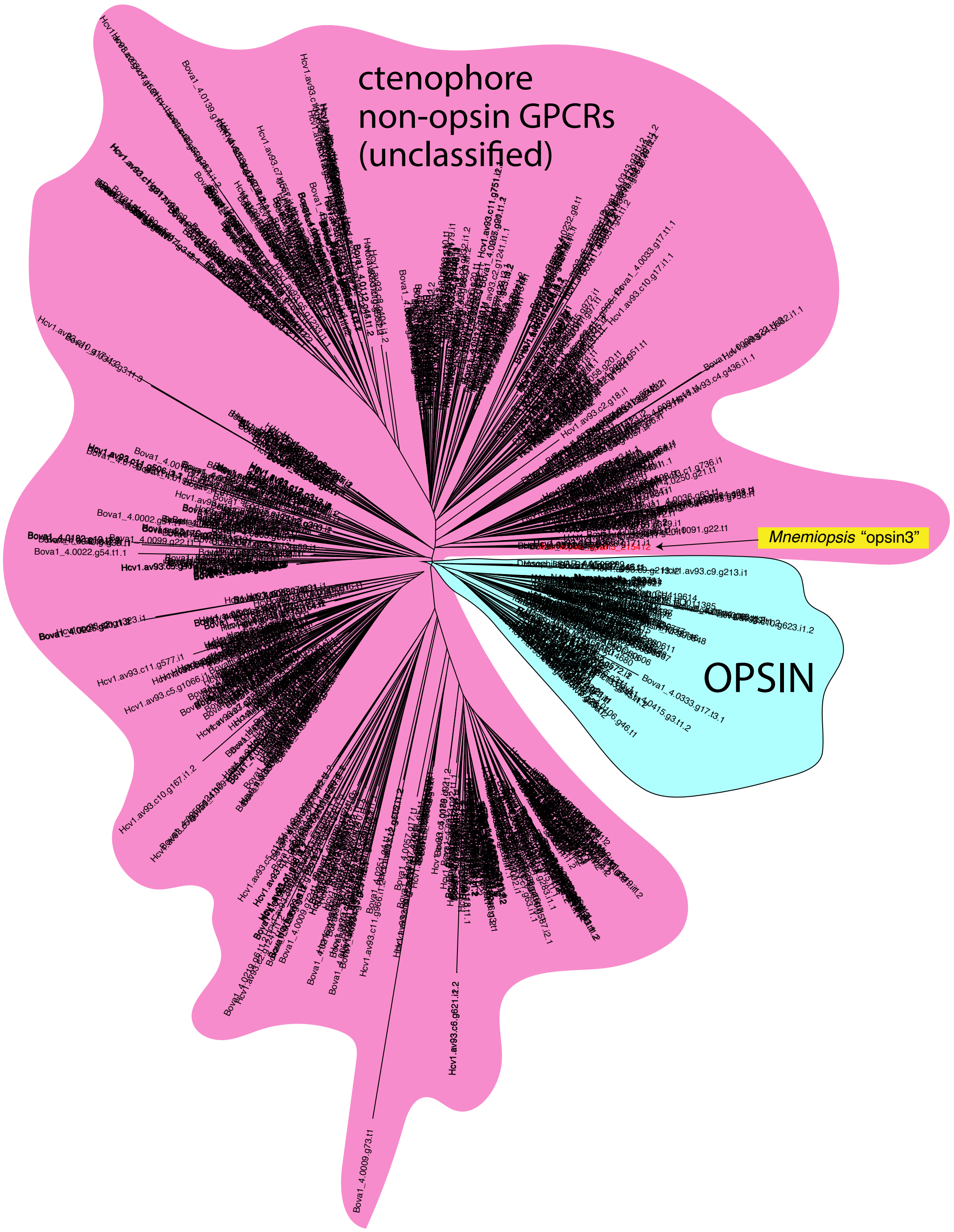

Figure S1. *Mnemiopsis* "opsin3" groups in a clade of ctenophore non-opsins. Taxa in pink are unclassified non-opsin GPCRs from *Beroe ovata* and *Hormiphora californensis*. The clade highlighted in blue consists of opsins from ctenophores, cnidarians, and bilaterians from Schnitzler et al. (2012) (10). The tree was estimated with a maximum likelihood analysis in IQ-TREE using the LG model.

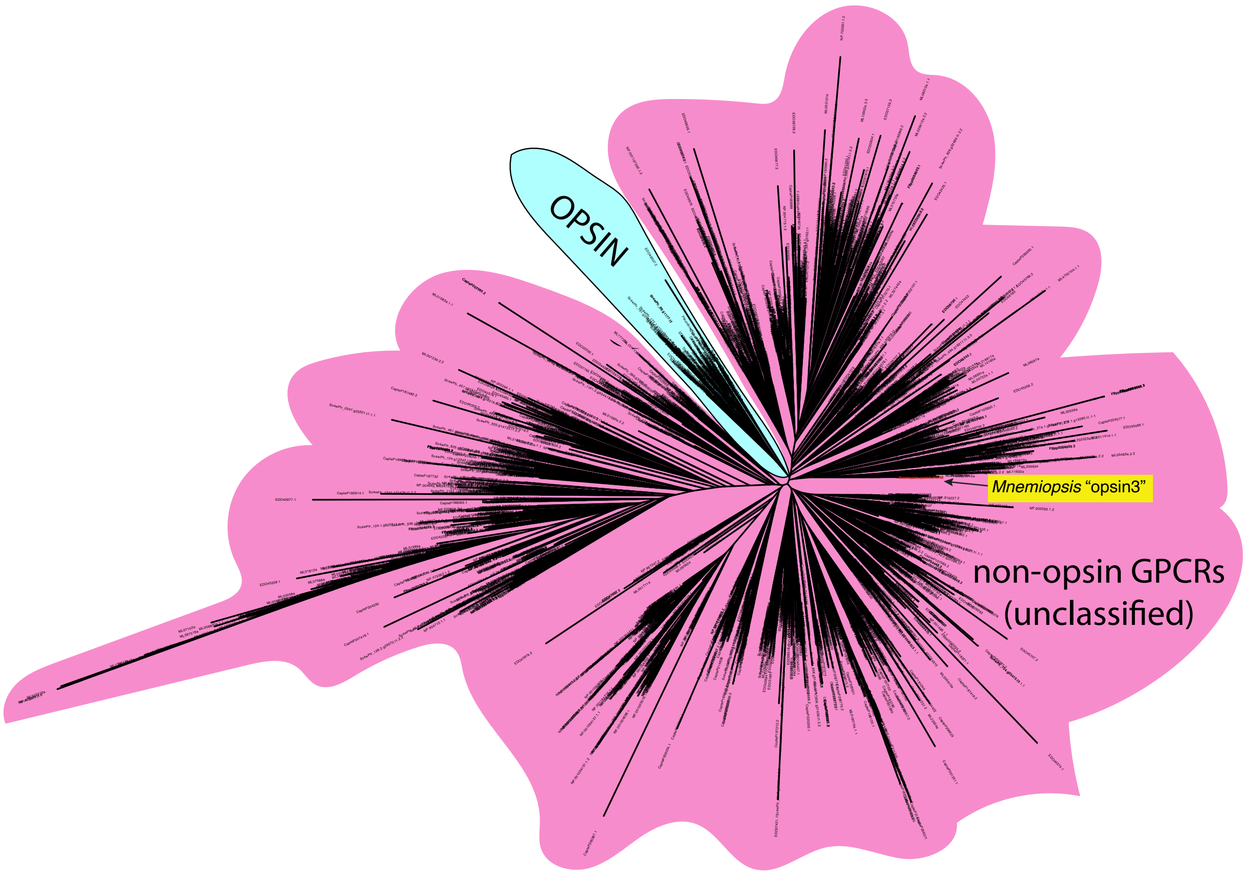

Figure S2. *Mnemiopsis* “opsin3” groups in a clade of ctenophore, cnidarian, and bilaterian non-opsins. Taxa in pink are unclassified GPCRs from *Mnemiopsis leidyi*, several cnidarians, and several bilaterians (see the detailed list of taxa in the Supplementary Analyses). The clade highlighted in blue consists of opsins from ctenophores, cnidarians, and bilaterians from Schnitzler et al. (2012) (10). The tree was reconstructed using a maximum-likelihood analysis in IQ-TREE with the LG model.

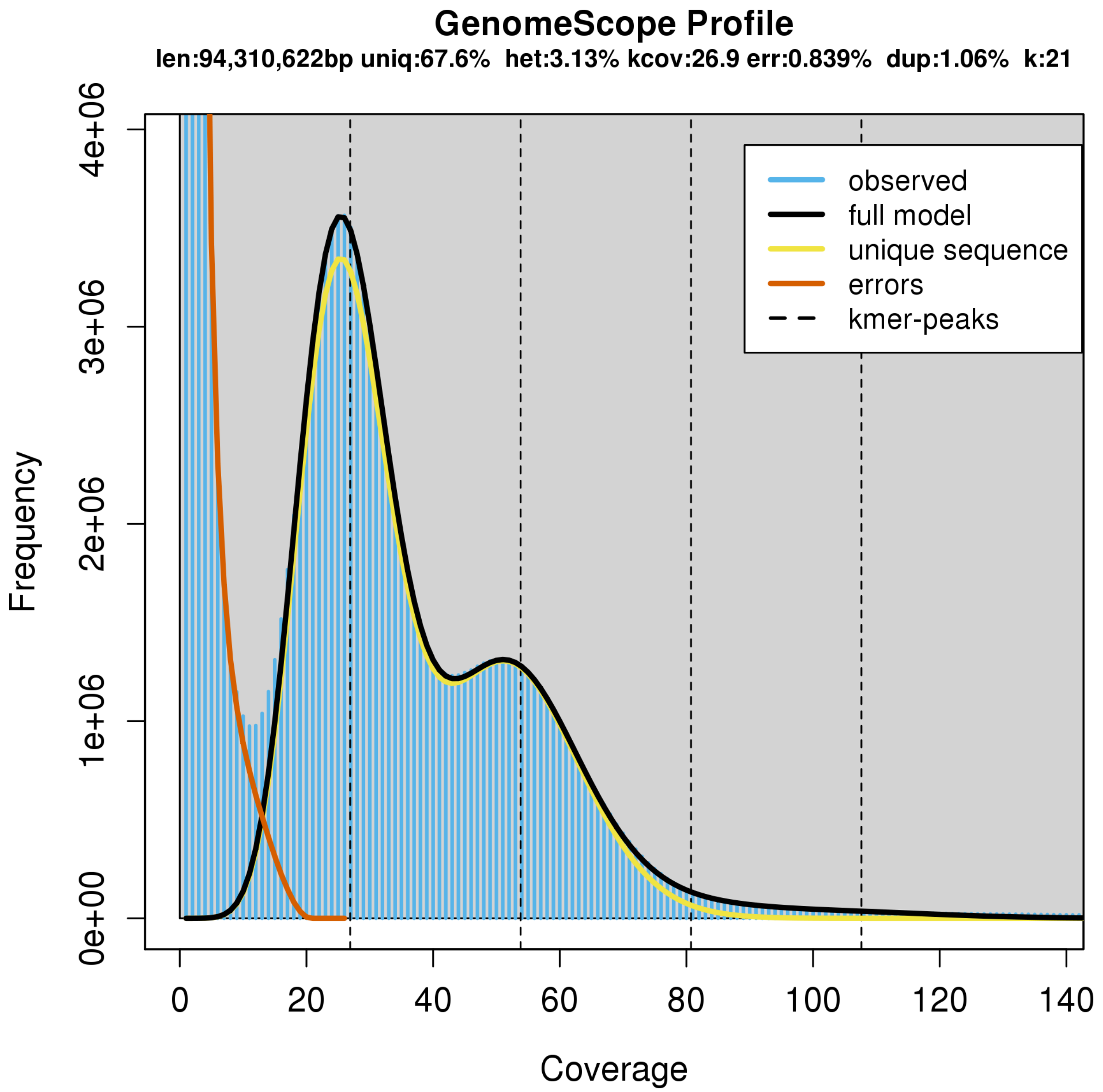

Figure S3. *Beroe ovata* genome size is estimated as 94.3 Mb. The genome size was estimated using GenomeScope. The histogram was generated using counts of canonical k-mers of k=21 for DNA sequenced from one individual using Illumina.

A Bova1_4.0066.g65

Features of Bova1_4.0066.g65

Scaffold	Type	Start	End	Strand	Transcript ID
Bova1_4.0066	gene	271610	273346	-	Bova1_4.0066.g65.t1
Bova1_4.0066	mRNA	271610	273346	-	Bova1_4.0066.g65.t1
Bova1_4.0066	exon	271610	272095	-	Bova1_4.0066.g65.t1
Bova1_4.0066	exon	272371	272513	-	Bova1_4.0066.g65.t1
Bova1_4.0066	exon	272667	272831	-	Bova1_4.0066.g65.t1
Bova1_4.0066	exon	272960	273050	-	Bova1_4.0066.g65.t1
Bova1_4.0066	exon	273317	273346	-	Bova1_4.0066.g65.t1
Bova1_4.0066	CDS	271610	272095	-	Bova1_4.0066.g65.t1
Bova1_4.0066	CDS	272371	272513	-	Bova1_4.0066.g65.t1
Bova1_4.0066	CDS	272667	272831	-	Bova1_4.0066.g65.t1
Bova1_4.0066	CDS	272960	273050	-	Bova1_4.0066.g65.t1
Bova1_4.0066	CDS	273317	273346	-	Bova1_4.0066.g65.t1
Bova1_4.0066	start_codon	273344	273346	-	Bova1_4.0066.g65.t1
Bova1_4.0066	stop_codon	271610	271612	-	Bova1_4.0066.g65.t1

```
>Bova1_4.0066.g65.t1
MIYVQQGILQAQLECQTQFSKNRWNCTSIDNNPFIKLLERGFRESAFSYAILSAGITYML
TNACGEGQFPECFCNNNLVDSYDAANSSDTFHFTNTSSWHMEGCDDNVEVASNMVMDAAP
SRRGRSSDTRDLVVKHNNEVGIKALQDTVTLDCRCHGMSGTCATKTCMRKMPPFSVVGDY
LQYKFYGARRVIQKKSGYGLLVASGSRREPREPRSDELLYYEKSPNFCAAEPAINWAGTS
GRECALTNDPRQRDSCEVLCCKRGFRTTVEEAVKDCNCRAKFEDYWSSMECDKCTVQQER
HFCR
```

```
>Bova1_4.0066.g65.t1
ATGATATACGTCCAGCAAGGCATCCTACAGGCTCAGCTGGAGTGTCAGACTCAGTTCAGT
AAAAACCGATGGAACTGTACTTCCATTGACAACAACCCCTTCATAAAACTTCTTGAGCGA
GGGTTCAGAGAGTCAGCATTCTCCTATGCCATCCTCTCCGCTGGGATAACGTACATGTTG
ACCAACGCTTGCGGAGAGGGACAGTTCCCAGAGTGTTTCTGTAACAATAACCTGGTGGAC
TCGTATGATGCCGCTAATTCATCAGATACTTTCCACTTCACTAACACAAGTTCGTGGCAT
ATGGAGGGGTGTGATGATAATGTTGAGGTGGCATCCAATATGGTCATGGATGCCGCGCCC
TCCAGACGAGGTCGTAGTAGTGACACACGGGACCTCGTGGTCAAGCATAACAATGAGGTC
GGCATCAAGGCACTCCAGGACACCGTCACACTGGACTGCAGGTGTCACGGCATGTCGGGC
ACGTGTGCCACCAAGACTTGTATGCGGAAAATGCCCCCTTTTTCCGTGGTGGGCGACTAC
CTGCAGTACAAATTCTACGGAGCACGGCGTGTCATACAGAAGAAGTCCGGTTACGGGTTG
CTCGTGGCGTCCGGTTCCCGGCGTGAGCCCCGCGAACCTCGGAGCGACGAGCTCCTCTAC
TACGAGAAGAGTCCGAACTTCTGTGCGGCAGAGCCGGCTATCAACTGGGCAGGGACATCA
GGACGAGAGTGTGCGTTGACTAACGATCCCAGGCAGCGTGATAGCTGTGAGGTGTTGTGC
TGTAAACGGGGGTTTCGCACCACGGTGGAGGAGGCTGTCAAGGATTGTAACTGTCGGGCT
AAATTCGAGGATTATTGGAGTAGTATGGAGTGTGATAAGTGTACAGTACAGCAGGAGAGA
CATTTCTGTAGGTGA
```

B

Bova1_4.0066.g65.t1 vs. Human RefSeq BLASTP

NP_003382.1	8.05e-57
NP_110388.2	1.48e-55
NP_078613.1	1.35e-51
NP_006513.1	1.44e-50
NP_003386.1	8.95e-50

Bova1_4.0066.g65.t1 vs. Uniprot BLASTP

WNT4_XENLA	1.07e-55
WNT2_HUMAN	1.46e-55
WNT4_CHICK	1.96e-55
WNT2_PONAB	2.30e-55
WNT2_PAPAN	2.30e-55

Bova1_4.0066.g65.t1 vs. Mnemiopsis leidyi BLASTP

ML101013a	7.69e-162
ML01051a	7.43e-36
ML06021a	1.19e-33
ML07528a	6.35e-24

Bova1_4.0066.g65.t1 vs. Hormiphora californensis BLASTP

Hcv1.av93.c3.g1180.i1	1.75e-92
Hcv1.av93.c5.g1084.i1	1.21e-36
Hcv1.av93.c5.g1084.i2	5.21e-36
Hcv1.av93.c5.g47.i1	1.13e-33
Hcv1.av93.c5.g47.i2	2.08e-08

PFAM domains detected in Bova1_4.0066.g65.t1

family	E-Value	Alignment start	Alignment end
wnt	2.1e-78	2	304

C

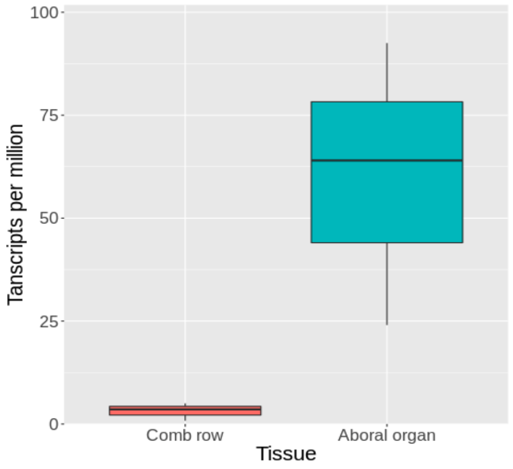

Figure S4. Data available on the *Beroe ovata* Database (BovaDB) gene page. To generate this figure, we queried Bova1_50066.g65 where A) features gene annotations and corresponding transcript sequences and B) displays BLASTP hits against the Human RefSeq database, UniProt database, *Mnemiopsis leidyi* protein models, and *Hormiphora californensis* protein models, as well as detected Pfam-A domains. The query also shows C) gene expression in transcripts per million for tissues sampled from the comb rows and aboral organ of *B. ovata*.

Supplementary Tables

Assembly	Residue count	Seq count	N50	BUSCO comp	BUSCO comp+part	BUSCO OPCG
Platanus k=31	102,602,585	256,545	1,831	58.43%	84.31%	1
Platanus k=45	110,194,112	342,834	1,215	56.47%	81.96%	1.01
Platanus k=63	104,680,639	304,253	1,022	45.88%	76.86%	1
Platanus k=31 scaffolded with k=45 & k=63	77,735,948	57,230	3,047	58.43%	83.92%	1
CANU size = 89 Mb	193,469,553	2,935	118,073	84.31%	94.51%	1.77
CANU size = 89 Mb correctedErrorRate (CER) = 0.075	197,427,515	2,793	131,137	84.31%	94.51%	1.82
CANU size = 89 Mb CER = 0.075 corOutCoverage (COC) = 200	211,712,022	3,324	130,089	84.71%	94.12%	1.87
CANU size = 120 Mb CER = 0.25 COC = 200	176,335,242	4,967	51,566	82.35%	94.51%	1.47
CANU size = 120 Mb CER = 0.30 COC = 200	155,065,417	4,318	57,432	81.96%	94.12%	1.42
Optimal Platanus k=31 scaffolded with CANU assemblies	159,704,438	22,153	520,768	72.16%	88.24%	1
Optimal Platanus k=31 (scaffolded with CANU) 5 kb gaps removed	100,157,706	25,280	187,267	70.98%	88.24%	1
Optimal Platanus k=31 (scaffolded with CANU) 5 kb gaps & redundancy removed	91,618,099	4,991	233,527	82.75%	92.94%	1.03

Table S1. Intermediate genome assembly statistics. The assembly column describes the assembler used for the data and corresponding parameters, where size is the estimated genome size, CER is the correctedErrorRate, and COC is the corOutCoverage parameters in Canu. BUSCO comp denotes the percentage of complete core genes detected against the eukaryotic database. BUSCO comp+part indicates the percentage of complete and partial genes detected against the eukaryotic database. BUSCO OPCG represents the average number of orthologs per core gene. The row highlighted in blue presents the statistics for the final assembly.

Gene ID	Log Fold Change	False discovery rate	Human RefSeq BLASTP	Accession no.	*M. leidyi* ortholog
Bova1_5.0075.g28	11.4307332	0.000621107	chromosome 9 open reading frame 85	NP_872311.2	ML008030a
Bova1_5.0064.g29	9.63557447	0.000752666	tubulointerstitial nephritis antigen	NP_055279.3	ML05234a
*Bova1_5.0086.g15	8.44396385	2.09E-05	nephrocystin 3	NP_694972.3	ML073030a
*Bova1_5.0211.g20	7.87650137	0.000919684	ciliary microtubule inner protein 1	NP_848551.1	ML159716a
Bova1_5.0089.g35	7.83493441	0.000130271	ankyrin 2	NP_001139.3	ML13779a
Bova1_5.0080.g11	7.72261703	2.98E-05	tripartite motif containing 71	NP_001034200.1	ML25772a
Bova1_5.0333.g3	7.6591059	2.99E-05	kelch like family member 20	NP_055273.2	ML078929a
Bova1_5.0038.g58	7.53729578	0.000435077	tripartite motif containing 45	NP_079464.2	ML15993a
Bova1_5.0042.g28	7.48636756	4.06E-05	tripartite motif containing 45	NP_079464.2	ML065727a
Bova1_5.0375.g7	7.42327503	0.000813359			ML147613a
Bova1_5.0252.g4	7.23997096	1.46E-05	creatine kinase, mitochondrial 1A	NP_001308855.1	ML216311a
Bova1_5.0203.g10	6.82556723	4.06E-05	uncharacterized protein	XP_038070611.1	ML142412a
Bova1_5.0157.g9	6.79318737	0.000254927	polycystin 2 like 1, transient receptor potential cation channel	NP_057196.2	ML01487a
Bova1_5.0003.g97	6.60560995	0.000501861	erbb2 interacting protein	NP_001240626.1	ML019227a
Bova1_5.0032.g111	6.35922224	3.76E-05	tubulin alpha 1c	NP_116093.1	ML01482a
Bova1_5.0392.g5	5.95152509	0.000290219			ML111727a
Bova1_5.0057.g7	5.68823768	3.73E-05	protein tyrosine phosphatase receptor type T	NP_008981.4	ML22523a
Bova1_5.0106.g11	5.59703513	2.99E-05	vicilin-like seed storage protein At2g18540	XP_001636586.1	ML16082a
Bova1_5.0203.g16	5.4795858	0.000202584			ML216322a
*Bova1_5.0091.g28	5.31873903	0.000731676	sperm associated antigen 8	NP_001034681.1	ML030212a
Bova1_5.0030.g65	5.00728496	0.000792607	hemicentin 1	NP_114141.2	ML146510a
*Bova1_5.0013.g96	4.96312812	0.000209047	RGP1 homolog, RAB6A GEF complex partner 1 (CTENO189)	NP_001073965.2	ML45843a
Bova1_5.0010.g78	4.95586074	0.000760298			ML049618a
Bova1_5.0042.g25	4.92147171	0.000264485	tripartite motif containing 71	NP_001034200.1	ML065725a
*Bova1_5.0092.g21	4.600518	2.99E-05	ciliary microtubule associated protein 1A	NP_444510.2	ML03453a
*Bova1_5.0074.g42	3.88084653	0.000394613	flagellar calcium-binding protein TB-44A-like	XP_057297192.1	ML00881a
Bova1_5.0162.g28	3.84184103	0.000209047			ML094323a
Bova1_5.0275.g8	3.54030524	0.000518427	LIM domain 7	NP_001293009.1	ML120740b
Bova1_5.0122.g29	3.42190212	9.80E-05	RCC1 domain containing 1	NP_291022.2	ML009613a

Bova1_5.0027.g6	3.38145849	0.000303993			ML13703a
Bova1_5.0004.g11	3.06650171	0.000919684	kelch like family member 3	NP_059111.2	ML20254a
*Bova1_5.0025.g66	2.95424516	0.000715982	cilia and flagella associated protein 299	NP_689983.2	ML083030a
Bova1_5.0278.g5	2.86497465	0.000314872	growth arrest specific 8	NP_001472.1	ML154185a
*Bova1_5.0039.g17	2.6832581	0.000760298	cilia and flagella associated protein 157	NP_001012520.2	ML00233a
*Bova1_5.0022.g12	2.47996053	0.000744657	coiled-coil domain containing 63	NP_689804.1	ML17379a
*Bova1_5.0036.g27	2.4764606	0.000663612	coiled-coil domain containing 65	NP_149115.2	ML27892a
*Bova1_5.0042.g60	2.43396248	0.000385823	sperm flagellar 2	NP_079143.3	ML02238a
*Bova1_5.0019.g96	2.2985397	0.000730171	radial spoke head component 4A	NP_001010892.1	ML03101a
Bova1_5.0113.g19	2.15479075	0.000597355	EF-hand domain containing 2	NP_079460.2	ML12704a
*Bova1_5.0020.g123	3.78253199	0.001098517	tektin 1	NP_444515.1	ML047948a
*Bova1_5.0019.g42	3.53214346	0.004671784	(CTENO64)		ML032211a
Bova1_5.0022.g84	3.40455313	0.003674419	solute carrier family 18 member B1	NP_439896.1	ML05363a
*Bova1_5.0082.g21	2.61262134	0.003286169	ciliary microtubule associated protein 2	NP_001104003.1	ML09684a
Bova1_5.0147.g25	2.53613187	0.00887823	dual specificity tyrosine phosphorylation regulated kinase 2	NP_006473.2	ML04281a
Bova1_5.0009.g75	2.23297271	0.002503474	leucine rich repeat containing 74A	NP_001372035.1	ML329911a
*Bova1_5.0002.g47	2.12140602	0.006880405	basal body orientation factor 1	NP_079333.2	ML046518a
*Bova1_5.0097.g35	1.96310234	0.001472194	sperm associated antigen 6	NP_036575.1	ML020047a
*Bova1_5.0206.g16	1.92755813	0.004238991	stabilizer of axonemal microtubules 2	XP_023566608.1	ML161314a
*Bova1_5.0195.g11	1.86003103	0.00330019	sperm tail PG-rich repeat containing 2	NP_777612.1	ML039714a
*Bova1_5.0003.g46	1.81322966	0.004188171	sperm microtubule associated protein 2 like	NP_001243404.1	ML116812a
Bova1_5.0335.g6	1.60113889	0.003084842	IQ motif containing H	NP_001026885.2	ML047313a
*Bova1_5.0009.g74	1.54124782	0.006913752	dynein axonemal heavy chain 12	NP_001352957.1	ML329912a
Bova1_5.0029.g12	1.48769179	0.005223019	AFG1 like ATPase	NP_660358.2	ML001920a
Bova1_5.0194.g34	1.40554348	0.005798963	tetratricopeptide repeat protein 6 isoform 1	NP_001297064.1	ML263524a
Bova1_5.0208.g6	1.32045875	0.003728573	X-ray radiation resistance associated 1	NP_001365086.1	ML14172a

Table S2. *Beroe ovata* conserved genes differentially expressed in the comb rows relative to the aboral organ. Genes in white had a false discovery rate of ≤ 0.001 and an absolute value of log2 fold change ≥ 2 in *B. ovata,* and a false discovery rate of ≤ 0.01 in *Mnemiopsis leidyi.* Genes in orange had a false discovery rate of ≤ 0.01 in *B. ovata,* and a false discovery rate of ≤ 0.001 with an absolute value of log2 fold change ≥ 2 in *M. leidyi*. The Human RefSeq BLASTP column shows top hits with an E-value < 0.001 from BLASTP searches against the Human RefSeq database. * represent genes associated with cilia and/or sperm.

Gene ID	Log Fold Change	False discovery rate	Human RefSeq BLASTP	Accession no.	*M. leidyi* ortholog
Bova1_5.0057.g17	-15.45451248	0.000597355	relaxin family peptide receptor 1	NP_067647.2	ML322212a
Bova1_5.0062.g47	-8.034014927	0.000539107			ML050827a
Bova1_5.0306.g1	-6.836758504	0.000496888	sushi, von Willebrand factor type A, EGF and pentraxin domain containing 1	NP_699197.3	ML35931a
Bova1_5.0055.g36	-6.115832126	0.000807469			ML088210a
Bova1_5.0101.g33	-5.394198929	0.000663612	epithelial cell transforming 2 like	NP_001071174.1	ML015739a
Bova1_5.0064.g51	-5.355238054	0.000281878	Wnt family member 7A	NP_004616.2	ML01051a
Bova1_5.0042.g4	-5.059495666	0.000215868			ML04208a
Bova1_5.0391.g4	-4.955627849	0.000715982	H6 family homeobox 3	NP_001099044.1	ML02215a
Bova1_5.0216.g7	-4.313841718	0.000837446	ciliary rootlet coiled-coil, rootletin	NP_055490.4	ML092622a
Bova1_5.0024.g77	-4.312854091	0.000147207	discs large MAGUK scaffold protein 4	NP_001308004.1	ML141725a
Bova1_5.0024.g99	-4.244750965	0.000715982	POU class 1 homeobox 1	NP_000297.1	ML18891a
Bova1_5.0168.g20	-4.18342497	0.000880532	synaptotagmin 2	NP_796376.2	ML02779a
Bova1_5.0002.g73	-3.911398829	0.000285914	myostatin	NP_005250.1	ML102235a
Bova1_5.0175.g10	-3.792411517	0.000819048	thyroid hormone receptor interactor 11	NP_004230.2	ML120748a
Bova1_5.0300.g4	-3.555193285	0.000394613	hemicentin 2	NP_001278744.1	ML004937a
Bova1_5.0128.g29	-3.370716743	0.000488705	myelin regulatory factor	NP_001120864.1	ML01568a
Bova1_5.0075.g46	-3.078642456	0.000209047	tetraspanin 7	NP_004606.2	ML210026a
Bova1_5.0163.g40	-3.041536526	0.000808036	erythrocyte membrane protein band 4.1 like 4B	NP_061987.3	ML050828a
Bova1_5.0015.g66	-3.016029216	0.000435077	regulatory factor X1	NP_002909.4	ML154187a
Bova1_5.0082.g1	-2.602966198	0.000510693	dynein axonemal heavy chain 5	NP_001360.1	ML04658a
Bova1_5.0019.g63	-2.416756548	0.000919684	ankyrin repeat and EF-hand domain containing 1	NP_071379.3	ML00487a
Bova1_5.0040.g7	-10.79902282	0.001859142	tolloid like 2	NP_036597.1	ML348710a
Bova1_5.0002.g36	-5.583966688	0.002168193	GREB1 like retinoic acid receptor coactivator	NP_001136438.1	ML27444a
Bova1_5.0004.g160	-5.024877569	0.003517748			ML05422a
Bova1_5.0020.g122	-4.750706132	0.00148598	Myb/SANT DNA binding domain containing 7	NP_001365714.1	ML047942a
Bova1_5.0017.g69	-4.686628656	0.002345895	solute carrier family 22 member 8	NP_004245.2	ML273222a
Bova1_5.0229.g7	-4.479791997	0.002535575	uncharacterized protein	XP_043895268.1	ML04339a

Bova1_5.0013.g93	-4.244972959	0.005842223	ankyrin repeat and ubiquitin domain containing 1	NP_001138432.1	ML45845a
Bova1_5.0189.g12	-3.155066485	0.006512544	interferon regulatory factor 2 binding protein 1	NP_056464.1	ML040524a
Bova1_5.0001.g169	-3.119575611	0.006481614	chromosome 4 open reading frame 17	NP_115525.2	ML45138a
Bova1_5.0265.g17	-2.929872526	0.002503474	arylsulfatase family member I	NP_001012301.1	ML02095a
Bova1_5.0389.g7	-2.855435767	0.002641181	Synaptic vesicle 2 (SV2) related protein	NP_061181.1	ML042713a
Bova1_5.0050.g30	-2.841765961	0.00106233	neurexin 1	NP_001317007.1	ML044119a
Bova1_5.0067.g25	-2.741730983	0.00680795			ML13536a
Bova1_5.0054.g11	-2.706186952	0.001475364	integrin subunit alpha 8	NP_003629.2	ML30811a
Bova1_5.0066.g6	-2.482421175	0.001347256	SIX homeobox 1	NP_005973.1	ML174734a
Bova1_5.0068.g8	-2.28800103	0.008465708	transcription factor 7 like 2	NP_001139746.1	ML11223a
Bova1_5.0252.g5	-2.261687627	0.006531293	calcium dependent secretion activator 2	NP_060424.9	ML131114a
Bova1_5.0069.g23	-2.108411124	0.002071475			ML13174a
Bova1_5.0121.g13	-1.963573135	0.00721033	protein tyrosine phosphatase receptor type N2	NP_002838.2	ML078935a
Bova1_5.0246.g11	-1.871082411	0.001773252	PKHD1 like 1	NP_803875.2	ML311619a
Bova1_5.0059.g26	-1.753275488	0.003464214	transient receptor potential cation channel subfamily C member 5	NP_036603.1	ML090215a
Bova1_5.0247.g9	-1.648287564	0.006392136	ciliary microtubule inner protein 2B	NP_001157782.1	ML15094a
Bova1_5.0015.g38	1.345090547	0.008342949	zinc finger CCHC-type containing 24	NP_699198.2	ML311630a
Bova1_5.0076.g50	4.422806396	0.003994686	tubulin polymerization promoting protein family member 3	NP_057048.2	ML28205a

Table S3. *Beroe ovata* conserved genes highly expressed in the aboral organ relative to comb rows. Genes shown in white had a false discovery rate of ≤ 0.001 with an absolute value of log2 fold change ≥ 2 in *B. ovata* and false discovery rate of ≤ 0.01 in *Mnemiopsis leidyi*. Genes in orange had a false discovery rate of ≤ 0.01 in *B. ovata* and a false discovery rate of ≤ 0.001 in *M. leidyi* with an absolute value of log2 fold change ≥ 2 in *M. leidyi*. The Human RefSeq BLASTP column displays top hits from BLASTP searches against the Human RefSeq database with an E-value < 0.001.

Phylum	Species	Abbreviation
Porifera	*Amphimedon queenslandica*	Aqu or Aq
	Oscarella lobularis	Olo
Placozoa	*Trichoplax adhaerens*	Tad or Td
Cnidaria	*Acropora millepora*	Ami
	Nematostella vectensis	Nve or Nv
	Podocoryne carnea	Pca
Annelida	*Capitella sp.*	Csp
	Capitella teleta	Cte
	Platynereis dumerilii	Pdu
Platyhelminthes	*Schistosoma japonicum*	Sja
Mollusca	*Aplysia californica*	Aca
	Biomphalaria glabrata	Bgl
	Crassotrea gigas	Cgi
	Lottia gigantea	Lgi
Arthropoda	*Apis mellifera*	Ame
	Archaearanea tepidariorum	Pte
	Bombyx mori	Bmi
	Drosophila melanogaster	Dme
	Tribolium castaneum	Tca or Tc
Nematoda	*Caenorhabditis elegans*	Cel
	Trichinella sp.	Tsp
Echinodermata	*Strongylocentrotus purpuratus*	Spu
Hemichordata	*Saccoglossus kowaleskii*	Sko
Chordata	*Branchiostoma floridae*	Bfl or Bf
	Ciona intestinalis	Cin
	Ciona savignyi	Cs
	Danio rerio	Dre
	Halocynthia roretzi	Hro
	Homo sapiens	Hsa or Hs
	Lethenteron japonicum	Lja
	Mus musculus	Mmu or Mm
	Xenopus laevis	Xla or Xl

Table S4. Taxa included in our phylogenetic analyses of TGF-β and Wnt gene families. Taxa and sequences are from Pang et al. (2011) (11) and Pang et al. (2010) (12)**.**

References

1. Haas,B.J., Papanicolaou,A., Yassour,M., Grabherr,M., Blood,P.D., Bowden,J., Couger,M.B., Eccles,D., Li,B., Lieber,M., *et al.* (2013) De novo transcript sequence reconstruction from RNA-seq using the Trinity platform for reference generation and analysis. *Nature Protocols 2013 8:8*, **8**, 1494–1512.
2. McGinnis,S. and Madden,T.L. (2004) BLAST: at the core of a powerful and diverse set of sequence analysis tools. *Nucleic Acids Res*, **32**, W20–W25.
3. Johnson,L.S., Eddy,S.R. and Portugaly,E. (2010) Hidden Markov model speed heuristic and iterative HMM search procedure. *BMC Bioinformatics*, **11**, 1–8.
4. Pett,W., Ryan,J.F., Pang,K., Mullikin,J.C., Martindale,M.Q., Baxevanis,A.D. and Lavrov,D. V. (2011) Extreme mitochondrial evolution in the ctenophore *Mnemiopsis leidyi* : Insight from mtDNA and the nuclear genome. *Mitochondrial DNA*, **22**, 130–142.
5. Bernt,M., Donath,A., Jühling,F., Externbrink,F., Florentz,C., Fritzsch,G., Pütz,J., Middendorf,M. and Stadler,P.F. (2013) MITOS: Improved de novo metazoan mitochondrial genome annotation. *Mol Phylogenet Evol*, **69**, 313–319.
6. Ryan,J.F. (2019) hmm2aln.pl.
7. Nguyen,L.T., Schmidt,H.A., Von Haeseler,A. and Minh,B.Q. (2015) IQ-TREE: A Fast and Effective Stochastic Algorithm for Estimating Maximum-Likelihood Phylogenies. *Mol Biol Evol*, **32**, 268–274.
8. Li,H. (2023) Protein-to-genome alignment with miniprot. *Bioinformatics*, **39**.
9. Ryan,J.F., Pang,K., Mullikin,J.C., Martindale,M.Q. and Baxevanis,A.D. (2010) The homeodomain complement of the ctenophore Mnemiopsis leidyi suggests that Ctenophora and Porifera diverged prior to the ParaHoxozoa. *Evodevo*, **1**, 1–18.
10. Schnitzler,C.E., Pang,K., Powers,M.L., Reitzel,A.M., Ryan,J.F., Simmons,D., Tada,T., Park,M., Gupta,J., Brooks,S.Y., *et al.* (2012) Genomic organization, evolution, and expression of photoprotein and opsin genes in Mnemiopsis leidyi: a new view of ctenophore photocytes. *BMC Biol*, **10**.
11. Pang,K., Ryan,J.F., Baxevanis,A.D. and Martindale,M.Q. (2011) Evolution of the TGF-β Signaling Pathway and Its Potential Role in the Ctenophore, Mnemiopsis leidyi. *PLoS One*, **6**, e24152.
12. Pang,K., Ryan,J.F., Comparative,N., Program,S., Mullikin,J.C., Baxevanis,A.D. and Martindale,M.Q. (2010) Genomic insights into Wnt signaling in an early diverging metazoan, the ctenophore Mnemiopsis leidyi. 10.1186/2041-9139-1-10.
